# Multiparametric Abdominal Ultrasound as a Source of Imaging Biomarkers in Predictive and Personalized Medicine: A Narrative Review

**DOI:** 10.3390/diagnostics16132045

**Published:** 2026-06-30

**Authors:** Simona Steliana Tudor, Ancuta Elena Tupu, Ionela Daniela Ferțu, Caterina Nela Dumitru, Claudia Simona Stefan

**Affiliations:** 1 Faculty of Medicine and Pharmacy, Medical-Pharmaceutical Research Center, “Dunarea de Jos” University of Galați, 800008 Galați, Romania; steliana.tudor@ugal.ro (S.S.T.); caterina.dumitru@ugal.ro (C.N.D.); claudia.stefan@ugal.ro (C.S.S.); 2 Clinical Hospital of Infectious Diseases “St. Venerable Parascheva”, 800179 Galați, Romania

**Keywords:** multiparametric ultrasound, imaging biomarkers, elastography, contrast-enhanced ultrasound, radiomics, artificial intelligence, personalized medicine

## Abstract

Multiparametric ultrasound (MPUS) has emerged as a clinically relevant platform for the non-invasive generation of quantitative imaging biomarkers, transforming conventional abdominal sonography from a morphological screening tool into a functional, data-rich instrument aligned with predictive and personalized medicine. This review aims to synthesize current evidence on MPUS-derived imaging biomarkers across the principal abdominal organ systems and to examine their integration with radiomics and artificial intelligence. A narrative structured review was conducted through searches of PubMed/MEDLINE, Scopus, and Web of Science (January 2007–May 2026), combining terms related to multiparametric ultrasound, elastography, contrast-enhanced ultrasound (CEUS), quantitative ultrasound, radiomics, and imaging biomarkers. Articles were selected purposively on the basis of relevance, methodological quality, and recency; the review does not follow PRISMA methodology and does not include a formal quantitative synthesis. MPUS integrates B-mode imaging, Doppler, elastography, CEUS, and quantitative acoustic parameters (attenuation coefficient, sound speed, and viscosity) within a single examination. In hepatology, multiparametric protocols enable non-invasive staging of steatosis, fibrosis, and steatohepatitis in metabolic dysfunction-associated steatotic liver disease, portal hypertension assessment, and focal lesion characterization. Emerging applications span pancreatic, renal, vascular, and digestive pathology. The integration of radiomics and artificial intelligence amplifies biomarker potential, enabling molecular subtype prediction, treatment-response assessment, and prognostic stratification. Multiparametric abdominal ultrasound holds genuine promise as a central, non-invasive platform in precision medicine. Standardization of protocols, multicenter validation of quantitative thresholds, and integration with clinical, biochemical, and molecular data remain the principal challenges limiting broader clinical translation.

## 1. Introduction

Medical imaging has undergone a profound transformation over the past two decades, evolving from predominantly morphological techniques toward functional, quantitative, and multiparametric modalities capable of characterizing tissue biology beyond structural anatomy. This shift has been driven by the convergence of advanced signal processing, digital image analysis, and machine learning, enabling the extraction of quantifiable parameters (imaging biomarkers), defined as objectively measured characteristics derived from medical images that serve as indicators of normal biological processes, pathogenic mechanisms, or responses to therapeutic interventions [1]. The systematic development and validation of imaging biomarkers have become a strategic priority in translational medicine, linking the visible phenotype of disease to its underlying molecular and biological substrate [2,3].

Within this landscape, ultrasound occupies a distinctive position among imaging modalities. It is the most widely deployed cross-sectional imaging technique globally, valued for its real-time capability, absence of ionizing radiation, low cost, portability, and bedside accessibility [4,5]. In abdominal medicine, ultrasound serves as the first-line diagnostic tool for a broad range of hepatic, pancreatic, renal, vascular, and digestive conditions. However, conventional B-mode ultrasound remains inherently limited in its ability to quantify tissue composition, characterize pathological processes at a biophysical level, or generate reproducible measurements suitable for longitudinal monitoring.

The concept of multiparametric ultrasound (MPUS) directly addresses these limitations by integrating multiple complementary acoustic interrogation techniques into a single, structured examination protocol [4,5]. MPUS combines conventional B-mode imaging with Doppler modalities for hemodynamic assessment, elastography for tissue stiffness quantification, contrast-enhanced ultrasound (CEUS) for perfusion analysis, and an expanding set of quantitative parameters (including attenuation coefficient, backscatter coefficient, sound speed, and viscosity) that interrogate the mechanical, vascular, and structural properties of tissues in a comprehensive manner [6,7]. Each technique generates distinct imaging biomarkers that, when interpreted in an integrated framework, provide a richer and more specific tissue characterization than any single modality alone [7,8].

The biomarker potential of MPUS encompasses a wide range of quantitative parameters: tissue stiffness values expressed in kilopascals, perfusion indices derived from time–intensity curves, attenuation coefficients reflecting hepatic fat content, viscosity measurements sensitive to tissue inflammation, and radiomic features encoding texture, heterogeneity, and spatial organization at sub-visual resolution [3,9,10]. Such parameters are inherently quantitative, amenable to standardization, and dynamically reflective of disease state, properties that align with the core requirements of predictive and personalized medicine, where individual patient trajectories must be anticipated and treatment strategies tailored accordingly [1,10].

Clinical evidence for MPUS-derived biomarkers has accumulated most extensively in hepatology. Multiparametric protocols combining elastography, attenuation imaging, and viscosity measurements have enabled non-invasive staging of steatosis, fibrosis, and inflammation in metabolic dysfunction-associated steatotic liver disease (MASLD), progressively reducing dependence on liver biopsy [5,11,12]. CEUS has established a robust role in the characterization of focal liver lesions, integrated within internationally endorsed reporting frameworks such as the CEUS Liver Imaging Reporting and Data System (LI-RADS) [13,14]. Beyond hepatology, emerging evidence supports the utility of MPUS in pancreatic mass characterization, acute pancreatitis severity assessment, renal mass differentiation, chronic kidney disease staging, inflammatory bowel disease monitoring, and vascular pathology including carotid plaque vulnerability and abdominal aortic aneurysm wall biology [15,16].

The integration of artificial intelligence (AI) and radiomics has substantially amplified the biomarker potential of MPUS. Radiomic feature extraction from elastography maps, CEUS sequences, and B-mode textures enables the construction of high-dimensional imaging signatures capable of predicting tumor genotype, treatment response, and long-term clinical outcomes beyond observer perception [2,17,18]. Machine learning and deep learning models trained on multiparametric ultrasound data have demonstrated diagnostic performance approaching or exceeding that of expert radiologists in specific tasks, signaling a transition from subjective interpretation toward objective, algorithm-assisted assessment [19,20,21]. These developments position MPUS not merely as a diagnostic tool, but as a platform for generating data-rich inputs to predictive clinical models [10,18].

Despite this progress, the clinical translation of MPUS as an imaging biomarker platform faces substantial challenges. Variability across ultrasound systems, heterogeneity in acquisition protocols, insufficient multicenter validation of quantitative thresholds, and the absence of unified reporting frameworks collectively constrain the reproducibility and interoperability of MPUS parameters [8,22,23]. The integration of imaging biomarkers with clinical, biochemical, and molecular data into robust, externally validated predictive models remains an active area of methodological development [1,10].

This narrative structured review provides a comprehensive synthesis of current evidence on multiparametric abdominal ultrasound as a source of imaging biomarkers, with emphasis on its implications for predictive and personalized medicine. We examine the physical principles and derived parameters of each MPUS component technique, review clinical applications across the major abdominal organ systems, analyze the role of radiomics and artificial intelligence in expanding the biomarker potential of MPUS, and discuss the standardization requirements, limitations, and future directions that will determine the extent to which MPUS fulfills its promise as a central platform in precision abdominal imaging.

## 2. Materials and Methods

This work is a narrative review and does not follow the PRISMA methodology for systematic reviews; accordingly, no formal quantitative synthesis, meta-analysis, or risk-of-bias assessment was performed. A structured literature search was conducted in PubMed/MEDLINE, Scopus, and Web of Science covering the period from January 2007 to May 2026, using combinations of the terms “multiparametric ultrasound”, “elastography”, “contrast-enhanced ultrasound”, “quantitative ultrasound”, “shear-wave”, “attenuation imaging”, “radiomics”, “artificial intelligence”, and “imaging biomarkers”, together with organ-specific terms (liver, pancreas, kidney, bowel, carotid, aorta). Eligible sources comprised original clinical and methodological studies, systematic reviews and meta-analyses, consensus statements, and clinical practice guidelines published in English. Studies were selected purposively rather than exhaustively, prioritizing methodological quality, clinical relevance, recency, and representativeness across abdominal organ systems and MPUS techniques; case reports, conference abstracts without full text, and non-English publications were not considered. Because the selection was purposive and non-systematic, the cited literature comprises a mixture of primary studies, guidelines, consensus documents, and review articles, and the reference set should therefore be interpreted as a representative rather than an exhaustive or unbiased sample of the available evidence. As is inherent to narrative reviews based predominantly on published literature, the synthesis may be subject to publication bias, with positive findings potentially over-represented relative to negative or inconclusive ones.

**Figure 1:**
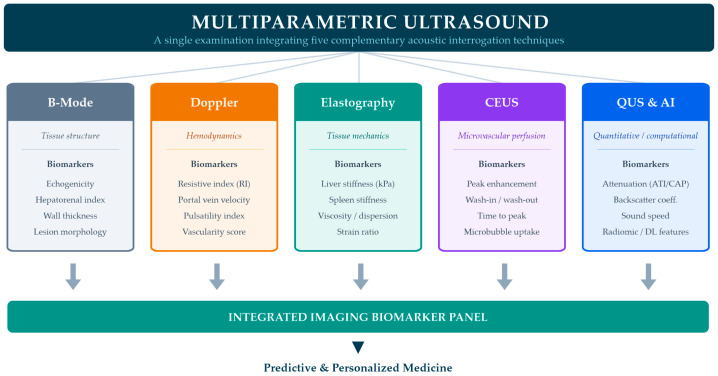
Components of Multiparametric Ultrasound and Derived Imaging Biomarkers.

The defining characteristic of MPUS as an imaging platform lies in its capacity to interrogate the same tissue from multiple acoustic perspectives within a single examination session, generating a panel of complementary biomarkers rather than a single qualitative assessment [4,5]. The integration of structural, hemodynamic, mechanical, and perfusion data within one protocol transforms ultrasound from a morphological screening tool into a quantitative instrument capable of supporting clinical decision-making at the level of diagnosis, risk stratification, and therapeutic monitoring [4,6,7]. The following subsections describe each component technique, its physical basis, and the imaging biomarkers it generates. The five principal MPUS components and their derived biomarkers are illustrated schematically in Figure 1.

### 2.1. B-Mode Ultrasound and Quantitative Parameters

B-mode ultrasound remains the structural foundation of any multiparametric protocol. It operates on the principle of pulse-echo interrogation: high-frequency acoustic pulses propagate through tissue, and the amplitude of backscattered signals, determined by acoustic impedance mismatches at tissue interfaces, is encoded as a grayscale image in real time. Spatial resolution depends on transducer frequency, focusing, and beam geometry, while tissue echogenicity reflects the aggregate acoustic properties of the interrogated volume.

Beyond morphological assessment, B-mode ultrasound provides the essential anatomical reference frame onto which all quantitative MPUS layers are superimposed. In the liver, increased parenchymal echogenicity relative to the renal cortex has historically been used as a qualitative surrogate for steatosis, although this approach carries significant inter-observer variability and limited sensitivity for mild fat infiltration [5,11]. Contemporary systems have incorporated quantitative B-mode-derived parameters, including the hepatorenal index and automated brightness analysis, as adjuncts to dedicated fat quantification tools [24].

In focal lesion characterization, B-mode features, such as lesion margins, internal architecture, posterior acoustic behavior, and parenchymal background, constitute the entry point for multiparametric lesion analysis, informing the subsequent application of CEUS and elastography [4,23].

### 2.2. Doppler Techniques

#### 2.2.1. Color and Power Doppler

Color Doppler imaging visualizes the spatial distribution of blood flow within a region of interest by encoding the mean frequency shift of backscattered signals as a color overlay on the B-mode image. Power Doppler, which encodes the amplitude rather than the direction of the Doppler signal, offers superior sensitivity for low-velocity and low-volume flow, at the cost of directional information. Both modalities are essential components of MPUS, enabling the semi-quantitative assessment of tissue vascularity, identification of pathological neovascularity in focal lesions, and evaluation of macrovascular patency in portal and hepatic venous systems.

In hepatology, color Doppler contributes to the assessment of portal hypertension through measurement of portal vein flow velocity, direction, and congestion index [25]. In inflammatory bowel disease, the Limberg scoring system classifies bowel wall vascularity on color Doppler as a marker of mucosal and transmural inflammation [16]. In oncology, the vascular pattern of a focal lesion, central versus peripheral, chaotic versus organized, provides initial characterization that is subsequently refined by CEUS [23].

#### 2.2.2. Spectral Doppler and Hemodynamic Indices

Spectral Doppler analysis quantifies blood flow velocity over time within a defined sample volume, generating waveform profiles from which hemodynamic indices are derived. The resistive index (RI = [peak systolic velocity − end-diastolic velocity]/peak systolic velocity) and pulsatility index (PI) reflect downstream vascular resistance and are measured routinely in hepatic, renal, and mesenteric arterial beds. Elevated RI values in the hepatic and splenic arteries correlate with increased sinusoidal resistance in chronic liver disease [25]. In renal applications, interlobar artery RI serves as a marker of parenchymal resistance, with values above 0.70 associated with reduced renal function and early fibrosis [15]. The 0.70 value represents an approximate threshold rather than a rigid cut-off, as the resistive index is physiologically influenced by age, heart rate, and systemic vascular comorbidity and should be interpreted within the clinical context of each patient. Spectral Doppler of the hepatic veins provides a qualitative and semi-quantitative index of right heart hemodynamics and sinusoidal compliance, with triphasic waveform flattening correlating with advancing hepatic fibrosis [25].

### 2.3. Elastography

Elastography encompasses a family of techniques that quantify tissue mechanical properties, primarily stiffness and, more recently, viscosity, by analyzing the propagation characteristics of mechanical waves generated within tissue. Stiff tissues, such as fibrotic liver parenchyma or malignant masses, propagate shear waves at higher velocities and exhibit greater resistance to deformation than soft, normal tissues [6,7,26]. The clinical implementation of elastography has been codified in consensus statements from major ultrasound societies, providing standardized acquisition criteria and validated thresholds for clinical decision-making [27,28].

A structured comparison of the principal elastography techniques is provided in Table 1.

#### 2.3.1. Transient Elastography (TE/FibroScan)

Transient elastography measures liver stiffness by generating a low-frequency (50 Hz) mechanical vibration at the skin surface and tracking the resulting shear wave propagation velocity using a dedicated pulse-echo transducer [29]. The derived liver stiffness measurement (LSM), expressed in kilopascals, correlates with the degree of hepatic fibrosis as classified histologically by the METAVIR scoring system. Validated thresholds using the M probe identify significant fibrosis (F ≥ 2) at approximately 7.9 kPa, severe fibrosis (F ≥ 3) at 10.3 kPa, and cirrhosis (F4) at 11.9–13.0 kPa, with diagnostic accuracy (AUROC) exceeding 0.85 for advanced fibrosis across multiple etiologies [8,29]. These cut-offs are specific to vibration-controlled transient elastography (VCTE/FibroScan, Echosens (Waltham, MA, USA)) with the M probe and are not numerically interchangeable with the kilopascal values produced by shear-wave elastography techniques, which rely on a different physical principle; optimal thresholds also vary modestly with underlying etiology and probe selection (M vs. XL). TE is a non-imaging technique and does not generate a real-time elastographic map; it requires a dedicated device and is subject to failure in approximately 5% of cases, predominantly in patients with high body mass index or narrow intercostal spaces [29]. The controlled attenuation parameter (CAP), measured simultaneously by FibroScan, quantifies ultrasonic attenuation as a surrogate for hepatic steatosis, with validated thresholds across three degrees of fat infiltration [30,33].

#### 2.3.2. Point Shear-Wave Elastography (p-SWE/ARFI)

Point shear-wave elastography (p-SWE), implemented as Acoustic Radiation Force Impulse (ARFI) technology on conventional ultrasound platforms, generates a localized shear wave at a user-defined region of interest using a focused acoustic push pulse [6,7]. The system measures shear wave propagation velocity (in m/s) or derives tissue stiffness in kPa within a small sample volume superimposed on the B-mode image. This integration with real-time imaging enables operator-guided placement of the measurement region, avoiding vascular structures, bile ducts, and heterogeneous areas [27,28]. p-SWE has demonstrated diagnostic performance equivalent to or exceeding transient elastography for hepatic fibrosis assessment, with the additional advantage of being applicable in obese patients and those with ascites [31,32]. A vendor-neutral “rule of four” framework has been proposed for interpreting p-SWE measurements across different ARFI-based systems, improving interoperability [28].

#### 2.3.3. Two-Dimensional Shear-Wave Elastography (2D-SWE)

Two-dimensional shear-wave elastography generates a color-coded stiffness map overlaid on the B-mode image in real time, providing spatial visualization of tissue mechanical heterogeneity across a defined region [26,32]. The system uses ultrafast plane wave compounding to track the propagation of multiple shear wave fronts simultaneously, producing quantitative stiffness values in kPa with high temporal and spatial resolution. 2D-SWE has been validated for hepatic fibrosis staging, demonstrating superior diagnostic accuracy compared to ARFI-based techniques for the diagnosis of significant fibrosis (F ≥ 2) in cohorts including patients with NAFLD and chronic hepatitis B [26,31]. The spatial stiffness map also enables identification of focal areas of heterogeneous rigidity, potentially reflecting coexisting focal lesions or regional fibrosis distribution. In pancreatic, renal, and bowel applications, 2D-SWE provides tissue rigidity assessment in organs where point measurements may be insufficient to capture spatial heterogeneity [15,32].

#### 2.3.4. Strain Elastography

Strain elastography quantifies relative tissue deformation in response to external or internal mechanical compression, producing a color map in which softer tissues appear green and stiffer tissues appear blue or red, depending on vendor encoding [6,7]. Unlike shear-wave techniques, strain elastography does not provide absolute stiffness values; it generates qualitative or semi-quantitative indices such as the strain ratio (lesion stiffness relative to surrounding tissue) and histogram-based parameters [6]. While operator-dependent compression variability limits reproducibility, strain elastography retains clinical utility in superficial organ evaluation, lymph node characterization, and bowel wall assessment in inflammatory bowel disease, where the distinction between fibrotic and inflammatory stenosis has therapeutic implications [16]. Its incorporation into AI-assisted analysis pipelines has substantially improved diagnostic consistency [34].

### 2.4. Contrast-Enhanced Ultrasound (CEUS)

#### 2.4.1. Contrast Agents and Acquisition Protocol

CEUS employs intravenously administered ultrasound contrast agents (UCAs), stabilized microbubble suspensions of an inert gas enclosed within a phospholipid or albumin shell, as intravascular tracers that enhance the acoustic backscatter signal exclusively within the vascular compartment [35,36]. The most widely used agent in Europe and Asia is sulphur hexafluoride-filled microbubbles (SonoVue/Lumason; Bracco, Milan, Italy); perflutren-based agents (Optison, Definity (Waterloo, ON, Canada)) are predominantly used in North America. Unlike iodinated CT contrast or gadolinium-based MRI contrast, UCAs do not undergo renal excretion, carry no nephrotoxic risk, and can be safely administered to patients with renal impairment [23,37]. The purely intravascular distribution of microbubbles, which do not diffuse into the interstitium, enables dynamic, real-time visualization of tissue microvascular perfusion with a temporal resolution unavailable to CT or MRI [23,36].

Acquisition employs low mechanical index (MI < 0.1) continuous imaging in contrast-specific mode, capturing arterial (10–45 s), portal venous (45–120 s), and late (>120 s) phases following bolus injection [35,38]. Standardized examination protocols, endorsed by WFUMB-EFSUMB guidelines, specify injection parameters, timing definitions, and interpretation criteria for hepatic and non-hepatic applications [35,36,38].

**Figure 2:**
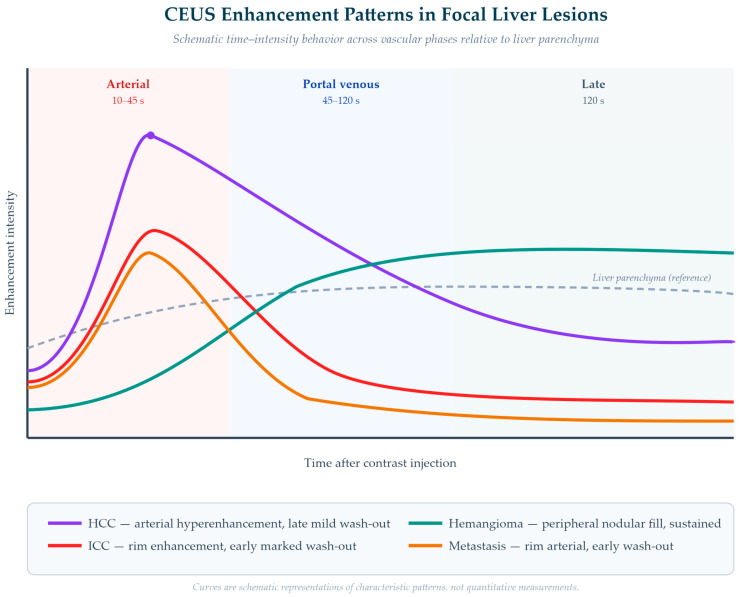
CEUS Enhancement Patterns in Focal Liver Lesions.

Characteristic enhancement patterns for the principal focal liver lesion types are illustrated schematically in Figure 2.

#### 2.4.2. Quantitative CEUS Parameters

Quantitative analysis of CEUS time–intensity curves (TIC), obtained via dedicated software platforms such as VueBox (Bracco (Milano, Italy)) or SonoLiver (Tomtec (Unterschleissheim, Germany)), extracts a panel of perfusion biomarkers from the dynamic enhancement curve of a defined region of interest [37]. Key derived parameters include: peak enhancement (PE), reflecting maximum microvascular filling; time to peak (TTP), representing vascular transit speed; wash-in rate (WiR) and wash-in area under the curve (WiAUC), reflecting inflow characteristics; mean transit time (MTT); and wash-out rate, which is particularly relevant for malignant hepatic lesion characterization [2,35,37]. Dynamic CEUS (D-CEUS) quantification has been applied to the differential diagnosis of primary liver tumors, demonstrating that PE and WiR are significantly higher in hepatocellular carcinoma compared to intrahepatic cholangiocarcinoma, supporting non-invasive lesion discrimination [15,23]. In pancreatic and renal applications, perfusion parameters derived from quantitative CEUS enable characterization of lesion vascularity as a surrogate for histological subtype and malignancy potential [15,38].

### 2.5. Quantitative Ultrasound and AI-Assisted Parameters

#### 2.5.1. Attenuation Coefficient, Backscatter, and Sound Speed

Quantitative ultrasound (QUS) techniques exploit the frequency-dependent attenuation and scattering of acoustic waves within tissue to derive parametric biomarkers of tissue composition [39,40]. The attenuation imaging coefficient (ATI or AI), expressed in dB/cm/MHz, measures the rate of ultrasonic energy loss per unit depth and frequency, reflecting the acoustic absorption and scattering properties of the interrogated tissue [30,33]. In the liver, elevated ATI values correlate with increasing degrees of steatosis, with proposed thresholds for S1 (≥0.60 dB/cm/MHz), S2, and S3 grades validated against controlled attenuation parameter and MRI-derived proton density fat fraction in multiple cohorts [24,30,33]. As attenuation-imaging implementations differ across manufacturers, these dB/cm/MHz thresholds are vendor-specific and not directly transferable between ultrasound platforms without system-specific validation. The backscatter coefficient captures the frequency-dependent amplitude of signals returning from tissue microstructure, providing complementary information to attenuation for characterizing hepatic parenchymal architecture [40]. Sound speed measurement, derived from the time-of-flight of acoustic pulses, varies with tissue density and compressibility; reduced sound speed values have been associated with hepatic fat accumulation, offering an additional non-invasive steatosis biomarker [24,39,41].

#### 2.5.2. Viscosity and Dispersion Parameters

Beyond elasticity, hepatic tissue exhibits viscoelastic mechanical behavior, meaning its response to dynamic loading depends on both elastic (stiffness) and viscous (energy-dissipation) components [41,42]. Viscosity measurement by shear-wave dispersion analysis, implemented as Vi.PLUS (Supersonic Imagine/Hologic (Aix-en-Provence, France)) or equivalent platforms, captures the frequency dependence of shear wave velocity, expressed as a dispersion slope in (m/s)/kHz. Higher viscosity values reflect tissue states characterized by cellular edema, inflammatory infiltration, and hepatocyte ballooning, with emerging evidence that Vi.PLUS improves the identification of metabolic steatohepatitis (MASH) beyond what elastography and attenuation alone can achieve [11,41,42]. Normative values in healthy liver parenchyma have been established across age groups [42], and combined multiparametric models incorporating viscosity, elasticity, and sound speed have demonstrated superior diagnostic performance for MASH compared to single-parameter approaches [11,41].

#### 2.5.3. AI-Derived Texture and Radiomic Features

The radiomic dimension of MPUS captures sub-visual tissue characteristics from all acoustic modalities (B-mode, elastographic maps, CEUS sequences, and QUS parametric images) through the systematic extraction of high-dimensional feature sets encoding intensity statistics, texture descriptors, morphological attributes, and wavelet-based characteristics [3,9,43]. These features can be extracted from raw radiofrequency data (H-scan analysis, Nakagami parameter, homodyned-K distribution) or from processed images using standardized pipelines aligned with the Image Biomarker Standardization Initiative [3,9,44]. AI-based models (including support vector machines, random forests, gradient boosting, and convolutional neural networks) then learn discriminative combinations of radiomic features to support diagnosis, molecular subtype prediction, prognosis, and treatment response assessment [43,44]. The application of deep learning directly to ultrasound radiofrequency data has further eliminated feature engineering requirements, enabling end-to-end learning from raw acoustic signals to clinical outputs [21,43,44]. In the context of MPUS, the multimodal nature of the input (combining elastographic, perfusion, and textural information) substantially expands the discriminative capacity of AI models compared to single-modality approaches [40,43].

The main MPUS-derived imaging biomarkers, their biological substrates, quantitative units, clinical applications, and evidence maturity are summarized in Table 2.

## 3. Clinical Applications in Abdominal Pathology

### 3.1. Hepatic Applications

The liver is the organ for which multiparametric ultrasound has achieved the most extensive clinical validation, driven by the global burden of chronic liver disease and the longstanding need for non-invasive alternatives to liver biopsy. MPUS protocols combining elastography, fat quantification, perfusion analysis, and viscosity measurement now enable comprehensive, non-invasive characterization of both diffuse parenchymal disease and focal lesions within a single examination session, positioning the liver as the model organ for multiparametric biomarker integration [4,5,41].

#### 3.1.1. Diffuse Liver Disease: Steatosis and Fibrosis Assessment in MASLD/NAFLD

Metabolic dysfunction-associated steatotic liver disease (MASLD), encompassing the former NAFLD/NASH spectrum, represents the most prevalent chronic liver condition globally, affecting an estimated 25–30% of the general population and constituting a leading cause of cirrhosis and hepatocellular carcinoma [12,53]. The clinical management of MASLD requires accurate, sequential assessment of three interconnected pathological processes: hepatic steatosis, fibrosis stage, and active steatohepatitis (MASH), each conferring distinct prognostic implications and guiding therapeutic decisions [12,53,54].

Liver biopsy has historically served as the reference standard for staging all three components, but its invasive nature, sampling error, and limited suitability for repeated assessments have driven the development of non-invasive testing strategies [54,55]. Serum-based indices such as FIB-4 and the NAFLD Fibrosis Score provide initial risk triage, but their diagnostic performance for individual patients is constrained by wide grey zones requiring confirmatory testing [56,57]. Elastography has emerged as the most robust non-invasive technique for fibrosis staging: liver stiffness measurement by vibration-controlled transient elastography (VCTE/FibroScan) achieves AUROC values of 0.85–0.88 for advanced fibrosis (F ≥ 3) and cirrhosis (F4) across NAFLD cohorts, outperforming serum markers when used in sequential algorithms [56,57]. Among shear-wave techniques, supersonic shear imaging (SSI/2D-SWE) demonstrated superior diagnostic accuracy compared to ARFI and FibroScan for significant fibrosis (F ≥ 2) in a prospective NAFLD cohort (AUROC 0.86 vs. 0.82 vs. 0.77), with the lowest failure rate among obese patients [58]. In a large individual patient data meta-analysis (*n* = 5735; LITMUS Consortium), sequential combinations of FIB-4 and LSM-VCTE reduced the need for liver biopsy to approximately 33% of patients while maintaining high sensitivity for advanced fibrosis, offering a cost-effective and clinically pragmatic strategy [57].

The quantification of hepatic steatosis by MPUS has advanced substantially with the development and validation of acoustic-based fat quantification tools. The controlled attenuation parameter (CAP), measured simultaneously with FibroScan, achieves diagnostic performance (AUROC 0.80–0.85) comparable to MRI-derived proton density fat fraction (MRI-PDFF), the current non-invasive reference standard for steatosis grading, across etiologically diverse cohorts [30,33,59]. On conventional ultrasound platforms, attenuation imaging (ATI/AI) and quantitative B-mode-derived backscatter parameters provide real-time fat estimation integrated within the B-mode examination, enabling simultaneous steatosis and fibrosis assessment without a dedicated device [24,41]. Multiparametric protocols combining ATI, sound speed, and shear-wave elastography within a single session have demonstrated the feasibility of comprehensive MASLD staging, including steatosis grade, fibrosis stage, and risk of MASH, in less than three minutes in the majority of patients [5,60].

The discrimination of MASH from simple steatosis represents the most diagnostically challenging frontier of non-invasive MASLD assessment, as histological necroinflammation and hepatocyte ballooning do not produce specific mechanical or acoustic signatures accessible to conventional elastography [11,12]. Viscosity measurement by shear-wave dispersion analysis (Vi.PLUS) has emerged as a promising biomarker for MASH activity: in a prospective cohort of 120 biopsy-confirmed MASLD patients, Vi.PLUS demonstrated good diagnostic performance for the detection of hepatocyte ballooning (AUROC 0.72), and a composite VAS-MASH-US score incorporating Vi.PLUS, aspartate aminotransferase, and sound speed achieved AUROC 0.75 for MASH diagnosis with 79% sensitivity, potentially reducing the need for liver biopsy in low-risk patients [11,42]. As these data derive from a single-center study, the reported cut-offs and performance metrics should be interpreted as preliminary and hypothesis-generating, pending confirmation in independent multicenter cohorts. Longitudinal MPUS monitoring in MASLD patients treated with lifestyle modification or pharmacological therapy enables repeated, non-invasive assessment of treatment response across all three pathological dimensions [61].

Normative reference data for elastographic and quantitative ultrasound parameters have been established in healthy adult and pediatric populations, providing the calibration benchmarks necessary for clinical interpretation [62]. The practical implementation of MPUS for MASLD in outpatient settings, including patients with increased skin-to-liver distance, a traditionally challenging population for elastography, has demonstrated completion rates exceeding 95% with reliable measurements in the majority of cases, confirming the broad applicability of the multiparametric approach [60].

#### 3.1.2. Portal Hypertension and Advanced Chronic Liver Disease

Clinically significant portal hypertension (CSPH), defined by a hepatic venous pressure gradient (HVPG) ≥ 10 mmHg, is the central driver of decompensation events in compensated advanced chronic liver disease (cACLD), including variceal hemorrhage, ascites, and hepatic encephalopathy [25]. Direct HVPG measurement remains the invasive reference standard but is available only in specialized centers, limiting its routine clinical application. Liver stiffness measurement by elastography has been established as a reliable non-invasive surrogate for CSPH: LSM values below 15 kPa reliably exclude CSPH in patients with viral hepatitis etiology, while values above 25 kPa strongly support its presence [25,28,32]. These VCTE-derived thresholds are best validated in viral and alcohol-related cACLD; their performance is more variable in MASLD-related disease, where adiposity and hepatic steatosis can influence stiffness measurements. The Baveno VII consensus recommendations integrate LSM with platelet count to identify patients with cACLD who can safely forgo endoscopic screening for high-risk varices, preventing unnecessary invasive procedures in a substantial proportion of patients [28,32].

Spleen stiffness measurement (SSM) by 2D-SWE has emerged as an independent and complementary biomarker of portal hypertension, reflecting the hemodynamic consequences of elevated portal pressure transmitted to splenic sinusoidal architecture [45,46]. A systematic review and meta-analysis of SSM by 2D-SWE demonstrated AUROC values of 0.87–0.91 for the prediction of high-risk esophageal varices requiring treatment, with a proposed cut-off of 53.25 kPa (100 Hz probe) enabling avoidance of upper endoscopy in a significant fraction of cirrhotic patients [45]. This threshold is specific to the dedicated 100 Hz spleen-stiffness acquisition and is not directly interchangeable with values obtained using standard liver-stiffness presets or other vendors’ systems; device-specific calibration is therefore required before clinical application. Hirooka et al. evaluated a multiparametric ultrasound protocol combining liver stiffness, spleen stiffness, and splenic volume index in patients with chronic liver disease, demonstrating that the integrated MPUS model outperformed individual parameters for the prediction of both portal hypertension severity and clinically relevant endpoints, including variceal progression and hepatic decompensation [46]. The combined liver-to-spleen stiffness ratio provides additional discrimination between cirrhosis and porto-sinusoidal vascular disease, conditions that share clinical presentation but differ in prognosis and management [45,46].

Doppler assessment of the portal and hepatic venous system provides hemodynamic context for elastographic findings: portal vein flow velocity below 15 cm/s, splenomegaly, and collateral vessel formation corroborate elastographic evidence of advanced portal hypertension [25]. The integration of Doppler indices, liver and spleen stiffness, and quantitative CEUS measurement of hepatic perfusion, capturing portal and arterial components of hepatic blood flow, within a single MPUS examination session represents the current frontier of non-invasive portal hypertension assessment [5,25,46].

#### 3.1.3. Focal Liver Lesions: Differential Diagnosis and Characterization

The non-invasive characterization of focal liver lesions (FLL) is one of the most clinically impactful applications of hepatic MPUS. CEUS, supported by international guidelines from WFUMB and EFSUMB, enables dynamic, real-time assessment of lesion vascularity across arterial, portal venous, and late phases, providing enhancement patterns that reflect the underlying vascular architecture and biological behavior of lesions [35,36]. The CEUS LI-RADS system, developed by the American College of Radiology, standardizes the reporting of focal liver lesions detected on CEUS in patients at risk for hepatocellular carcinoma (HCC), assigning probability categories (LR-1 through LR-5 and LR-M) based on enhancement pattern, wash-out timing, and lesion size [13]. The LR-5 category, characterized by arterial phase hyperenhancement and late/mild wash-out, carries a positive predictive value exceeding 95% for HCC in cirrhotic patients, permitting non-invasive diagnosis without tissue confirmation in appropriately selected patients [13,23].

The differential diagnosis between HCC and intrahepatic cholangiocarcinoma (ICC) represents a particularly relevant diagnostic challenge, as both arise in cirrhotic livers and share morphological overlap on B-mode imaging while differing fundamentally in prognosis and treatment [47]. Ainora et al. prospectively evaluated a multiparametric US score combining dynamic CEUS parameters and 2D-SWE in a cohort of 82 histologically confirmed HCC and ICC patients, demonstrating that peak enhancement on D-CEUS, hepatic cirrhosis status, and SWE value were independent predictors of histological diagnosis, with the integrated MP-US score achieving AUROC 0.836 for non-invasive discrimination, potentially avoiding liver biopsy in a significant proportion of cases [47]. It should be noted, however, that these findings originate from a single-center cohort of limited size; the proposed MP-US score therefore represents a promising but preliminary tool that requires external, multicenter validation before it can be regarded as an established diagnostic threshold. The rationale underlying this discrimination lies in the distinct vascular biology of HCC (arterial hyperenhancement reflecting VEGF-driven neoangiogenesis) versus ICC (peripheral rim enhancement, early wash-out reflecting desmoplastic stroma with central fibrosis), and the differential tissue stiffness profiles reflecting hepatic fibrosis background versus intratumoral desmoplasia [23,47].

For the characterization of smaller incidental lesions, particularly subcentimeter findings in non-cirrhotic patients, MPUS enables lesion-level discrimination that B-mode imaging cannot achieve. CEUS demonstrates sensitivity exceeding 95% for the detection and characterization of hemangiomas, focal nodular hyperplasia, focal fat deposition and sparing, and simple cysts, eliminating the need for CT or MRI in a substantial proportion of cases with indeterminate B-mode findings [35,37]. The US LI-RADS system extends standardized ultrasound-based reporting to include surveillance populations, integrating B-mode and CEUS findings within a unified probability framework [63].

#### 3.1.4. Hepatocellular Carcinoma: Treatment Monitoring, Surveillance, and Molecular Characterization

CEUS has established a validated role in the post-treatment assessment of HCC following locoregional therapies, thermal ablation (radiofrequency, microwave), transarterial chemoembolization (TACE), and radioembolization [14,48]. Complete ablation is defined on CEUS as the absence of any residual arterial phase hyperenhancement within the treated zone, with a sensitivity of 84–92% and specificity of 96–99% for residual viable tumor, broadly comparable to contrast-enhanced CT and MRI while offering immediate post-procedural feedback without radiation exposure [48]. A recent meta-analysis of CEUS-guided microwave ablation in HCC demonstrated that combined imaging and serum biomarker assessment at 4–6 weeks post-ablation significantly improved the prediction of local tumor progression compared to either approach alone, supporting the integration of CEUS within multiparametric treatment response protocols [48]. The updated CEUS LI-RADS Treatment Response Assessment (TRA) framework, incorporating non-radiation modalities, provides standardized criteria for the categorization of treatment response across all ablative and embolic locoregional strategies [14].

Beyond morphological response assessment, MPUS-derived radiomic and AI-based approaches are beginning to address the molecular characterization of HCC, an area of major unmet clinical need given the therapeutic implications of tumor biological subtype. Machine learning models combining ultrasound radiomic features with clinical data demonstrated AUROC 0.846 for the prediction of TP53 mutation status in a retrospective cohort of 154 HCC patients, outperforming clinical-only and radiomics-only models and establishing non-invasive molecular subtyping as a feasible clinical goal [64]. Integrated ultrasound radiomics incorporating B-mode texture and CEUS perfusion characteristics predicted PD-1 blockade efficacy in unresectable HCC, with the multiparametric model achieving significantly better predictive accuracy than single-modality approaches [65]. Deep learning models applied to combined B-mode and CEUS radiomic signatures enabled pre-operative prediction of the macrotrabecular-massive (MTM) HCC subtype, a histological variant associated with particularly aggressive behavior and poor prognosis, with an AUROC of 0.812 in an external validation cohort [66]. At the population level, AI-assisted analysis of multiparametric abdominal ultrasound data has demonstrated promising performance for the detection of MASLD-related liver fibrosis and early hepatic malignancy, with meta-analytic evidence suggesting that AI integration improves diagnostic accuracy beyond standard imaging interpretation [67].

A summary of key clinical studies evaluating MPUS in liver disease, including study design, patient populations, techniques employed, and principal diagnostic performance metrics, is provided in Table 3.

**Figure 3:**
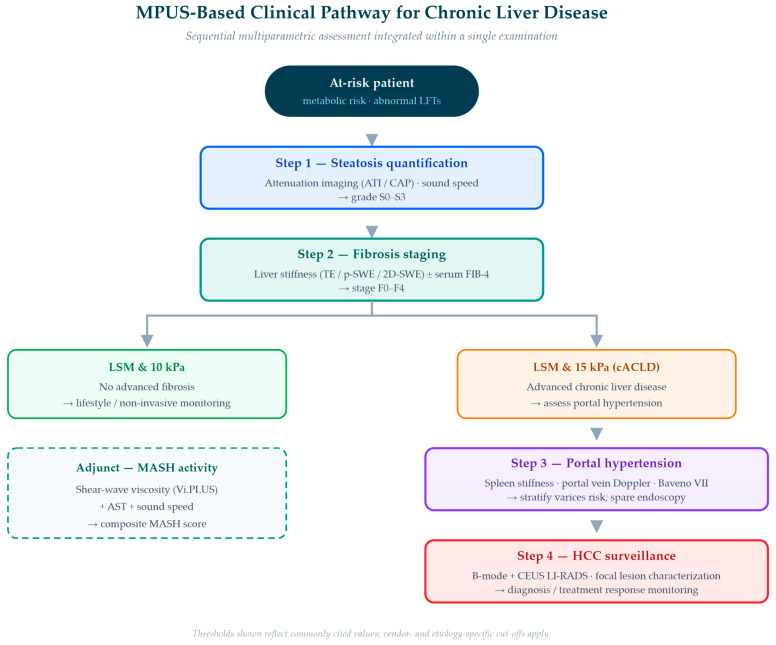
MPUS-Based Clinical Pathway for Chronic Liver Disease.

An integrated MPUS-based clinical management pathway for chronic liver disease is proposed in Figure 3.

### 3.2. Pancreatic Applications

The pancreas presents unique challenges for ultrasound-based assessment; its retroperitoneal location, adipose tissue coverage, and frequent obscuration by bowel gas impose technical constraints on transcutaneous imaging. Nevertheless, advances in ultrasound technology, including harmonic imaging, high-frequency probes, and microbubble-based contrast, have progressively expanded the role of multiparametric ultrasound in pancreatic diagnosis, complementing cross-sectional imaging with real-time, radiation-free, and bedside-accessible assessment [15,38].

#### 3.2.1. Acute Pancreatitis: Severity Stratification

Acute pancreatitis (AP) spans a clinical spectrum from self-limited interstitial edematous disease to necrotizing pancreatitis with organ failure and mortality exceeding 30% in its most severe forms. The revised Atlanta Classification (2012) introduced a standardized, clinically actionable severity framework distinguishing mild, moderately severe, and severe AP based on organ failure duration and the presence of local complications, pancreatic and peripancreatic necrosis, pseudocysts, and walled-off necrosis, providing the diagnostic and prognostic infrastructure within which imaging biomarkers must be interpreted [68].

Contrast-enhanced CT remains the reference standard for severity staging, necrosis quantification, and complication identification in AP. However, the nephrotoxic risk of iodinated contrast, ionizing radiation exposure, and the requirement for patient transport limit its applicability in the early stages of severe disease and in patients with renal impairment. CEUS has demonstrated diagnostic accuracy for pancreatic necrosis detection and severity grading broadly comparable to contrast-enhanced CT, with sensitivity of 91%, specificity of 100%, and a strong correlation between CEUS severity index and CT severity index (r = 0.926), Balthazar grade (r = 0.884), and extent of necrosis (r = 0.893) [69]. Quantitative CEUS analysis of pancreatic parenchymal perfusion distinguishes viable parenchyma (with normal wash-in dynamics) from inflammatory peri-pancreatic tissue (moderately enhanced) and necrotic zones (absent or markedly delayed enhancement), enabling a CEUS-based severity index directly analogous to the CT Severity Index [69,70]. CEUS can be performed at the bedside without nephrotoxic risk, making it particularly valuable as a follow-up imaging modality in patients with established CT staging at admission, and for the sequential monitoring of local complication evolution, including enlargement of walled-off necrosis collections or development of pseudoaneurysm, without additional radiation burden [15,70].

B-mode ultrasound, despite limited diagnostic accuracy for necrosis detection due to intestinal gas interference, retains an essential role in the initial evaluation of AP etiology, particularly in identifying gallstones, biliary sludge, and common bile duct dilation as triggers for biliary pancreatitis, and in monitoring peri-pancreatic fluid collections with Doppler to exclude vascular complications [68,69]. The integration of CEUS with B-mode and Doppler within a structured multiparametric protocol provides a comprehensive, repeatable, and radiation-free severity assessment pathway that the EFSUMB guidelines endorse as a clinically appropriate complement to CT-based staging [15,38].

#### 3.2.2. Pancreatic Masses and Cystic Lesions

The differential diagnosis of solid pancreatic lesions (SPL), encompassing pancreatic ductal adenocarcinoma (PDAC), neuroendocrine neoplasms (PanNEN), autoimmune pancreatitis, and metastatic deposits, critically determines management, as radical surgery, parenchyma-sparing strategies, and conservative follow-up carry fundamentally different risk-benefit profiles. Transcutaneous CEUS has established a diagnostically relevant role in SPL characterization based on the vascular architecture of lesions relative to surrounding pancreatic parenchyma [70].

PDAC, which constitutes approximately 85% of solid pancreatic neoplasms, exhibits characteristic hypoenhancement on CEUS across all phases, reflecting its desmoplastic stroma with minimal tumor vascularity. In prospective cohorts, the sign of hypovascularity as a CEUS criterion for PDAC achieves sensitivity of 90%, specificity of 100%, and accuracy of 93.8%, while the complementary sign of iso- or hypervascularity identifies non-ductal adenocarcinomas, predominantly PanNEN and serous adenoma, with sensitivity of 100% and specificity of 90% [70]. Quantitative CEUS perfusion analysis further characterizes the hemodynamic differences between PDAC and inflammatory masses such as pseudotumoral chronic pancreatitis, where preserved parenchymal perfusion distinguishes inflammatory from malignant hypoenhancement [70,71]. The application of CEUS immediately following the B-mode detection of a pancreatic mass within the same session, without requiring patient transfer or scheduling a separate CT/MRI, has been recommended by EFSUMB guidelines as a clinically efficient strategy to promote faster diagnosis and reduce diagnostic delay in PDAC, a malignancy where early detection is the primary determinant of resectability and survival [15,70].

Tissue stiffness assessed by elastography provides complementary characterization of SPL: pancreatic carcinoma and chronic pancreatitis exhibit significantly higher stiffness compared to normal pancreatic parenchyma, while PanNEN typically presents with lower stiffness values [71]. Multiparametric endoscopic ultrasound (EUS), combining B-mode EUS with CE-EUS, EUS elastography, and microvascular imaging, provides superior spatial resolution for small SPL not adequately visualized by transcutaneous imaging, with diagnostic performance for malignancy approaching 90% sensitivity and specificity in expert centers [71]. The integration of deep learning into EUS-based analysis has further improved lesion classification accuracy, with AI-assisted models achieving AUROC values of 0.87–0.93 for the discrimination of malignant from benign SPL in multicenter validation cohorts [71].

For pancreatic cystic lesions (PCL), a heterogeneous group including serous and mucinous cystadenomas, intraductal papillary mucinous neoplasms (IPMN), and pseudocysts, CEUS contributes to risk stratification by characterizing mural nodules, septal enhancement, and communication with the main pancreatic duct, features that correlate with high-grade dysplasia and malignant transformation [15,38]. The EFSUMB non-hepatic guidelines endorse CEUS as a valuable adjunct to CT and MRI for the follow-up of PCL, particularly for the dynamic characterization of mural nodule vascularity, which is not reliably captured by CT due to timing constraints [15].

### 3.3. Renal Applications

The kidney represents a clinically important target for multiparametric ultrasound assessment, encompassing two distinct diagnostic paradigms: the characterization of focal renal masses, where non-invasive differentiation of malignant from benign lesions directly guides surgical and surveillance decisions, and the longitudinal evaluation of diffuse parenchymal disease, where the non-invasive quantification of renal fibrosis and functional deterioration may reduce dependence on repeated biopsy [15,38].

#### 3.3.1. Renal Mass Characterization

The incidental detection of renal masses has increased substantially over the past two decades, driven by the expanding utilization of cross-sectional abdominal imaging for unrelated indications. It is estimated that more than half of individuals over the age of 50 harbor at least one undetermined renal lesion detectable on imaging, a proportion of which represents clinically significant malignancy requiring intervention [72]. Renal cell carcinoma (RCC), the predominant malignant renal neoplasm, encompassing clear cell, papillary, and chromophobe histological subtypes, carries distinct prognostic implications and management pathways that ideally should be anticipated pre-operatively. The European Association of Urology guidelines acknowledge the diagnostic role of CEUS for the characterization of indeterminate solid and cystic renal lesions, particularly in patients with contraindications to iodinated CT contrast or gadolinium-based MRI agents [73].

CEUS holds a distinctive advantage in renal mass evaluation precisely because ultrasound contrast agents are purely intravascular and undergo pulmonary elimination without renal excretion, rendering CEUS safe in patients with chronic kidney disease, renal failure, or renal transplantation, a population in which CT contrast nephrotoxicity and gadolinium-related nephrogenic systemic fibrosis represent clinically relevant concerns [15,38]. Dynamic CEUS characterization of solid renal masses exploits the differential vascular architecture of renal tumor subtypes: clear cell RCC typically demonstrates rapid, intense arterial hyperenhancement with early wash-out, reflecting its hypervascular nature and VEGF-driven neoangiogenesis; angiomyolipoma (AML) and oncocytoma show variable enhancement patterns that partially overlap with malignant subtypes, limiting but not eliminating discriminative capacity; papillary RCC, the second most common subtype, characteristically exhibits hypoenhancement relative to cortex across all phases, attributable to its poor vascularity [72,74]. A prospective study analyzing CEUS features of 287 renal tumors stratified by size demonstrated that the discriminative power of CEUS between malignant and benign lesions varies significantly with tumor diameter, with smaller lesions (<2 cm) showing greater enhancement pattern overlap and requiring integrated multiparametric interpretation [74].

For cystic renal lesions, CEUS improves upon B-mode and CT-based Bosniak classification by enabling real-time, high-sensitivity detection of fine septal enhancement, small mural nodules, and irregular wall thickening, features that predict malignant potential and guide surveillance intervals or surgical referral [72]. Multiple comparative studies have confirmed that CEUS sensitivity and specificity for the classification of complex cystic renal lesions are broadly comparable to MRI, while offering the additional advantages of real-time assessment without the spatial resolution constraints imposed by MRI respiratory motion [72].

Ultrasound elastography for solid renal mass characterization remains at an earlier stage of clinical validation compared to hepatic applications. Shear-wave velocity measurements in renal tumors are confounded by the depth- and position-dependent anisotropy of the renal parenchyma, inter-lesion stiffness variability attributable to histological heterogeneity, and the influence of renal blood flow on wave propagation [75,76]. Preliminary studies suggest that SWE-derived stiffness values and machine learning-based integration of multiple elastographic parameters may contribute to RCC-AML discrimination, but multicenter validation with larger cohorts and standardized acquisition protocols is required before clinical implementation can be recommended [72,76]. The emergence of multiparametric renal mass evaluation, integrating B-mode morphology, power Doppler vascularity, CEUS enhancement kinetics, and elastographic stiffness within a single examination, mirrors the multiparametric MRI paradigm that has transformed prostate cancer diagnosis, and represents a promising direction for non-invasive renal oncological assessment [72,73].

#### 3.3.2. Chronic Kidney Disease and Renal Fibrosis

Chronic kidney disease (CKD) affects approximately 10% of the global population and constitutes a major cause of morbidity, cardiovascular mortality, and end-stage renal disease requiring dialysis or transplantation. Regardless of primary etiology, diabetic nephropathy, hypertensive nephrosclerosis, glomerulonephritis, or obstructive nephropathy, the final common histopathological pathway is tubulointerstitial fibrosis, leading to progressive nephron loss and functional decline [75,76]. Accurate staging of renal fibrosis is essential to guide therapeutic intensity, predict progression trajectory, and assess response to nephroprotective interventions, yet renal biopsy, the histological reference standard, carries procedural risks, is subject to sampling error in heterogeneous disease, and is unsuitable for the repeated assessments required in longitudinal monitoring [75].

Shear-wave elastography has emerged as the most extensively evaluated non-invasive technique for renal parenchymal stiffness quantification, with the underlying premise that fibrosis-related extracellular matrix deposition increases tissue mechanical rigidity measurable by SWE [75,76]. A prospective cohort study enrolling 162 CKD patients who underwent renal biopsy demonstrated a significant positive correlation between cortical SWE values and histological fibrosis grade (r = 0.61, *p* < 0.001), with SWE outperforming serum creatinine and estimated glomerular filtration rate for the identification of severe fibrosis [76]. These results derive from a single-center cohort; renal SWE cut-offs remain insufficiently standardized across vendors and acquisition protocols to be applied as universal thresholds. A meta-analysis synthesizing data from multiple SWE studies in CKD confirmed that cortical stiffness values are significantly elevated in moderate and advanced CKD compared to healthy controls, with SWE demonstrating acceptable pooled sensitivity and specificity for the detection of significant fibrosis, though with heterogeneity attributable to variability in acquisition protocols, probe frequencies, and patient populations [75]. Importantly, renal SWE values are influenced not only by fibrosis but also by renal perfusion, hydration status, and vascular resistance, confounders that necessitate concurrent Doppler assessment of interlobar artery resistive index (RI) and careful clinical contextualization to avoid misclassification [75,76]. In keeping with this multifactorial dependence, renal cortical stiffness has been reported to increase in patients with congestive heart failure even when renal function is preserved or only mildly impaired, reflecting the contribution of venous congestion to measured stiffness and supporting a role for renal SWE as a non-invasive biomarker in systemic, cardiorenal conditions beyond primary parenchymal kidney disease [77].

Viscoelastic approaches combining shear-wave velocity with viscosity measurement have demonstrated added value for the concurrent assessment of renal fibrosis grade and interstitial inflammation: in a prospective study of 47 CKD patients with biopsy correlation, combined viscoelastic parameter models incorporating mean elasticity and mean viscosity by 2D-SWE achieved superior diagnostic performance for both fibrosis and inflammation grading compared to elasticity alone, supporting the premise that multiparametric mechanical characterization captures independent dimensions of renal histopathology [76]. This was a small, single-center exploratory study, and the added value of combined viscoelastic parameters requires validation in larger biopsy-controlled cohorts.

Doppler assessment of interlobar artery RI, an established biomarker of renal vascular resistance and parenchymal damage, contributes an independent hemodynamic dimension to renal MPUS evaluation, with RI values above 0.70 correlating with reduced glomerular filtration rate and accelerated CKD progression [25]. The integration of RI with SWE-derived stiffness and quantitative CEUS perfusion parameters, capturing renal cortical microvascular flow dynamics, within a structured multiparametric renal protocol represents a clinically feasible and diagnostically enriched approach to CKD staging that warrants prospective validation in dedicated multicenter cohorts [15,76].

### 3.4. Other Abdominal and Vascular Applications

Beyond the liver, pancreas, and kidney, multiparametric ultrasound has demonstrated expanding clinical applicability across abdominal vascular structures, the digestive tract, and associated soft tissue compartments. While the evidence base for these applications is less mature than that for hepatic disease, the consistent demonstration of additive diagnostic value across MPUS components, relative to B-mode alone, establishes a compelling rationale for multiparametric protocols in these domains [15,38].

#### 3.4.1. Abdominal Vascular Pathology

Conventional Doppler ultrasound has long served as the primary non-invasive tool for the assessment of arterial stenosis, plaque burden, and hemodynamic indices in abdominal and peripheral vessels. The integration of CEUS and elastography within vascular ultrasound protocols, constituting multiparametric vascular assessment, has substantially extended the diagnostic scope beyond luminal stenosis quantification to encompass plaque biology, wall inflammation, and adventitial neovascularization [78].

In the evaluation of carotid atherosclerotic disease, the identification of vulnerable or high-risk plaques, characterized histologically by a large lipid core, thin fibrous cap, intraplaque hemorrhage, and neovascularization, is of paramount clinical importance given that plaque vulnerability is the principal determinant of cerebrovascular ischemic events independent of stenosis severity. A prospective study of 43 patients undergoing carotid endarterectomy evaluated a multiparametric US protocol combining color Doppler, CEUS, and 2D-SWE against histological reference in the same surgical specimens [49]. CEUS demonstrated sensitivity of 87.1% and specificity of 58.3% for plaque vulnerability detection, with the key advantage of visualizing intraplaque neovascularization in real time through microbubble enhancement of vasa vasorum, while SWE provided complementary stiffness information reflecting fibrous cap integrity, with softer stiffness values associating with histological criteria of instability [49]. The multiparametric combination improved overall plaque risk classification compared to either technique alone, supporting the incorporation of CEUS and elastography alongside standard Doppler in the risk stratification of patients with carotid disease [49]. A broader review of vascular MPUS across carotid atherosclerosis, abdominal aortic pathology, and endovascular surveillance confirmed that the integration of CEUS, 3D ultrasound, and elastography into routine vascular imaging protocols enhances both diagnostic confidence and clinical decision-making across multiple vascular beds [78].

For abdominal aortic aneurysm (AAA), CEUS has demonstrated superior diagnostic accuracy over conventional color Doppler for the detection of endoleaks following endovascular aneurysm repair (EVAR), a critical surveillance endpoint, as persistent endoleak drives aneurysm sac pressurization and rupture risk. CEUS sensitivity for endoleak detection is comparable to CT angiography while avoiding ionizing radiation and nephrotoxic contrast, making it a preferred surveillance modality for long-term post-EVAR follow-up [78]. In the assessment of native AAA, quantitative CEUS characterization of adventitial microbubble uptake, reflecting inflammatory neovascularization within the aortic wall, has emerged as a promising imaging biomarker of aneurysm wall biological activity and growth rate: a prospective cohort study demonstrated that microbubble uptake in the aortic wall was present in 61% of patients with AAA ≥ 4 cm, and was associated with abnormal plasma inflammatory biomarkers, suggesting a mechanistic link between CEUS-detectable wall inflammation and accelerated aneurysm expansion [50]. This paradigm, using CEUS as a biomarker of wall biology rather than merely luminal patency, exemplifies the transition from morphological to functional vascular imaging that characterizes the multiparametric approach [50,78].

#### 3.4.2. Digestive Tract, Lymph Nodes, and Spleen

Intestinal ultrasound (IUS) has undergone a significant evolution in the assessment of inflammatory bowel disease (IBD), transitioning from a morphological technique measuring bowel wall thickness to a multiparametric platform integrating B-mode, color Doppler, CEUS, and shear-wave elastography within a single examination protocol [16,79,80]. This multiparametric intestinal US (MPIUS) approach captures distinct and complementary pathological dimensions of IBD activity: bowel wall thickness and layer stratification reflect transmural structural integrity on B-mode; the Limberg vascularity score on color Doppler quantifies mural hyperemia as a surrogate for active inflammation; CEUS time–intensity curve analysis provides objective, quantitative measurement of microvascular perfusion within the bowel wall, with peak enhancement and area under the curve correlating with histological inflammatory activity; and SWE-derived bowel wall stiffness distinguishes predominantly fibrotic from predominantly inflammatory stenoses, a clinically critical distinction, as fibrotic strictures require endoscopic or surgical intervention while inflammatory strictures may respond to intensified pharmacological therapy [16,79].

A systematic narrative review of MPIUS in IBD confirmed that the combination of CEUS and elastography with conventional IUS substantially improves the accuracy of disease activity assessment and therapeutic monitoring in Crohn’s disease, with the multiparametric approach achieving AUROC values of 0.92–0.95 for the differentiation of fibrotic from inflammatory strictures, significantly exceeding the performance of single-technique assessments [79]. In a prospective study of adult Crohn’s disease patients receiving biologic therapy, MPIUS parameters, including bowel wall thickness, Limberg score, SWE stiffness, and CEUS transmural enhancement pattern, demonstrated significant and concordant changes following treatment response, with normalization of CEUS enhancement preceding B-mode wall thickness reduction, suggesting that CEUS may serve as an earlier and more sensitive marker of mucosal healing than morphological parameters alone [16]. The integration of AI into intestinal ultrasound analysis is at an early but promising stage, with deep learning models applied to IUS video sequences demonstrating reproducible activity scoring independent of operator experience, potentially addressing one of the principal barriers to wider IUS adoption [80].

Beyond inflammatory disease, MPUS has demonstrated utility in the characterization of small intestinal neoplasms, including gastrointestinal stromal tumors (GISTs), carcinoid tumors, lymphomas, and adenocarcinomas, where the integration of B-mode morphology, CEUS enhancement pattern, and Doppler vascularity enables lesion-level risk stratification and guides the urgency of endoscopic or cross-sectional imaging referral [81]. For soft tissue masses adjacent to abdominal organs, including retroperitoneal sarcomas, desmoid tumors, and lymph node conglomerates, a comprehensive MPUS protocol combining B-mode architecture, Doppler flow mapping, CEUS perfusion characteristics, and elastography stiffness achieves diagnostic characterization that approaches the performance of MRI in expert hands, with the advantage of real-time guidance for biopsy targeting [82,83]. Lymph node CEUS characterization, differentiating reactive lymphadenopathy from malignant infiltration based on enhancement pattern, perfusion index, and afferent/efferent vascular architecture, further extends the biomarker repertoire of multiparametric abdominal ultrasound into the lymphatic compartment [82].

The spleen, previously discussed in the context of portal hypertension assessment (Section 3.1.2), contributes additional biomarker information within a comprehensive abdominal MPUS protocol: splenic volume, stiffness, and Doppler indices collectively reflect hemodynamic and parenchymal consequences of systemic diseases including hematological malignancies, storage disorders, and portal venous hypertension, and their integration within MPUS examinations enriches the diagnostic yield without requiring additional examination time [46,79].

The main non-hepatic applications of multiparametric ultrasound across pancreatic, renal, vascular, digestive, and lymphatic pathology are summarized in Table 4, highlighting the MPUS techniques used, the corresponding imaging biomarkers, and their principal clinical relevance.

## 4. Radiomics and Artificial Intelligence in Multiparametric Ultrasound

### 4.1. From Imaging Features to Imaging Biomarkers: The Radiomics Framework

Radiomics, the high-throughput extraction of quantitative features from medical images through automated computational analysis, represents a systematic methodology for converting visual information into mineable, high-dimensional data structures that can be interrogated for diagnostic, prognostic, and predictive signals [2,3]. The foundational premise is that medical images encode biological information at a spatial scale and complexity that exceeds human perceptual capacity: sub-visual patterns of texture, intensity distribution, morphological irregularity, and spatial heterogeneity may reflect underlying tissue microarchitecture, cellular density, extracellular matrix composition, and vascular organization, all features that bear direct relevance to disease phenotype and behavior [2,3,9].

**Figure 4:**
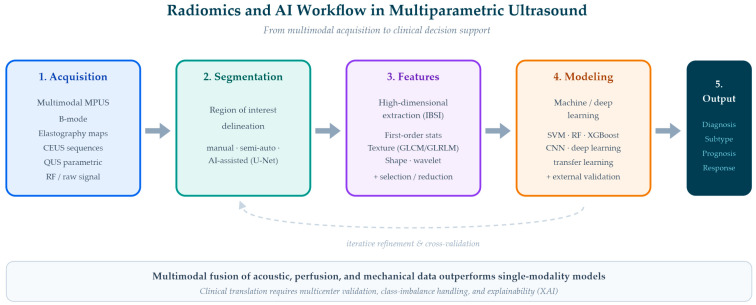
Radiomics and AI Workflow in Multiparametric Ultrasound.

The complete radiomics and AI analytical workflow is depicted in Figure 4.

The radiomics pipeline encompasses several sequential analytical stages: image acquisition under standardized protocols; preprocessing including normalization, noise reduction, and resampling; lesion or organ segmentation either manually, semi-automatically, or via AI-assisted delineation; feature extraction generating hundreds to thousands of quantitative descriptors per image, including first-order statistics (mean, entropy, skewness), shape and size metrics, texture features (GLCM, GLRLM, GLSZM matrices), and wavelet-based multi-scale characteristics; feature selection to identify the most informative and non-redundant subset; and model construction linking the radiomic signature to a clinical endpoint [9,10,51]. Standardization of feature definitions across imaging systems and software platforms is addressed by the Image Biomarker Standardization Initiative (IBSI), which has established harmonized protocols for feature computation to ensure reproducibility and cross-study comparability [9,51].

In the context of ultrasound, radiomics has been applied across the full spectrum of MPUS modalities, B-mode texture, elastographic maps, CEUS time–intensity sequences, and quantitative parametric images, with a rapidly growing body of evidence across hepatic, pancreatic, renal, thyroid, breast, and gastrointestinal applications [51]. A bibliometric analysis of ultrasound radiomics publications identified an exponential growth in the field between 2017 and 2023, with hepatocellular carcinoma, thyroid nodules, and breast lesions as the most frequently studied targets, and machine learning classification as the dominant analytical approach [51]. This trajectory reflects the broader adoption of radiomics as a translational research tool and signals its progressive transition from experimental investigation toward clinical decision support.

### 4.2. Machine Learning and Deep Learning Applications

#### 4.2.1. Supervised Models for Classification and Staging

Supervised machine learning, in which algorithms learn discriminative decision boundaries from labeled training data, constitutes the dominant analytical paradigm in ultrasound radiomics, applied to tasks including lesion classification, disease staging, molecular subtype prediction, and treatment response assessment [44,52,84]. Canonical supervised algorithms applied to MPUS-derived radiomic features include logistic regression, support vector machines (SVM), random forests, gradient boosting (XGBoost), and naïve Bayes classifiers, each offering distinct trade-offs between interpretability, computational efficiency, and predictive performance [44,52,84].

In hepatic oncology, machine learning models combining ultrasound radiomic features with clinical variables have demonstrated increasing sophistication in molecular characterization tasks that extend well beyond morphological description. An XGBoost-based combined model integrating radiomic features from B-mode ultrasound with clinical parameters achieved an AUROC of 0.846 for the prediction of TP53 mutation status in 154 HCC lesions, outperforming clinical-only and radiomics-only models [64]. A multiparametric approach combining B-mode and CEUS radiomic signatures in a deep learning framework enabled pre-operative identification of the macrotrabecular-massive HCC subtype, associated with aggressive biology and inferior prognosis, with an AUROC of 0.812 on external validation [66]. Integrated ultrasound radiomics incorporating perfusion parameters from quantitative CEUS predicted PD-1 blockade efficacy in unresectable HCC with significant prognostic discrimination, demonstrating the potential of MPUS-derived biomarkers to guide immunotherapy patient selection [65].

Beyond hepatic applications, supervised ML has been applied across multiple organ systems interrogated by multiparametric ultrasound. In prostate cancer detection, a nomogram based on multiparametric ultrasound radiomic features, integrating B-mode, SWE, and CEUS-derived descriptors, demonstrated significant discriminative capacity for malignant versus benign prostate lesions, providing a non-invasive pre-biopsy risk stratification tool [85]. For gastrointestinal stromal tumors, an AI-radiomics model trained on B-mode ultrasound and clinical data achieved accurate diagnosis and risk stratification, demonstrating generalizability across lesion subtypes [86]. In rectal cancer, deep learning applied to contrast-enhanced transrectal ultrasound radiomic signatures predicted distant metastasis with high accuracy, enabling non-invasive preoperative staging with potential to guide surgical planning [87]. In breast oncology, CEUS-derived radiomic models demonstrated robust performance for predicting axillary lymph node metastasis in a multicenter study, with the multiparametric radiomic signature outperforming conventional clinical and imaging predictors [88]. Quantitative ultrasound radiomic features extracted from conventional B-mode data predicted pathological complete response to neoadjuvant chemotherapy in breast cancer, a clinically pivotal endpoint, with diagnostic performance comparable to MRI-based models [89].

#### 4.2.2. Deep Learning and Convolutional Neural Networks

Deep learning, particularly convolutional neural networks (CNNs) trained end-to-end on image data, has introduced a paradigm shift in medical image analysis by eliminating the need for explicit feature engineering: the network learns hierarchical representations directly from raw pixel or voxel data, automatically identifying the spatial patterns most discriminative for the target task [52,84]. In ultrasound imaging, CNNs have been applied to B-mode images, elastographic maps, CEUS cine sequences, and multi-channel multiparametric inputs, achieving state-of-the-art performance across classification, segmentation, and regression tasks [52,84].

The application of deep learning to multiparametric ultrasound reaches its highest diagnostic performance when multiple acoustic modalities are combined as parallel input streams or fused at intermediate network layers. A quantitative 3D multiparametric ultrasound deep learning classifier incorporating CEUS-derived perfusion features and SWE stiffness maps achieved ROC AUC of 0.87 on internal validation and 0.88 on external validation for clinically significant prostate cancer detection, performance that is competitive with multiparametric MRI-based detection models [21]. Deep learning applied to SWE-derived elastographic maps of the liver, combined with B-mode texture features in a multicentre prospective cohort (*n* > 300 patients with chronic hepatitis B), achieved AUROC values of 0.93–0.97 for significant fibrosis and cirrhosis, substantially outperforming conventional LSM thresholds and radiologist interpretation, and has been prospectively validated across independent institutional cohorts [19]. Transfer learning strategies, which adapt networks pre-trained on large natural image datasets to medical imaging tasks, have substantially reduced the data requirements for deep learning model training in ultrasound applications, enabling high performance even in moderate-sized clinical cohorts [52,84].

### 4.3. AI-Assisted Elastography and Texture Analysis

The integration of AI specifically within elastographic workflows addresses one of the principal limitations of conventional elastography: the subjective, operator-dependent interpretation of qualitative strain maps and the inherent variability in shear-wave measurement quality under suboptimal acquisition conditions [34,43]. AI-based quality assessment algorithms now evaluate SWE measurement reliability in real time, flagging acquisitions affected by patient motion, inadequate coupling, or anatomical confounders, and automated segmentation tools delineate the region of interest for elastographic sampling without operator input, reducing inter-observer variability [34,43].

A systematic review and meta-analysis of AI-assisted ultrasound elastography for breast lesion classification demonstrated that AI-augmented elastographic analysis achieved a pooled sensitivity of 87.3% and specificity of 89.1% for malignant versus benign differentiation, significantly superior to unaided elastographic interpretation, with deep learning models incorporating both B-mode and elastographic inputs consistently outperforming single-modality approaches [90]. In thyroid nodule evaluation, combined AI analysis of B-mode texture, SWE stiffness maps, and CEUS enhancement patterns has improved the specificity of malignancy risk stratification beyond what conventional ACR TIRADS scoring achieves, potentially reducing unnecessary fine-needle aspiration biopsies in low-risk nodules [91,92]. The convergence of AI and elastography within MPUS frameworks, where stiffness maps serve simultaneously as clinical biomarkers and as AI input features, exemplifies the synergistic relationship between advanced image acquisition and computational analysis that defines modern multiparametric imaging [34,43,90].

### 4.4. Multimodal Integration and Clinical Validation

The full biomarker potential of MPUS in predictive and personalized medicine is realized not through individual technique optimization but through the principled integration of heterogeneous acoustic, perfusion, mechanical, and radiomic data streams into unified predictive models [1,10,17,18]. This multimodal integration paradigm, analogous to the multiparametric MRI approach that transformed prostate and abdominal oncological imaging, constructs patient-specific risk profiles from complementary information dimensions that together capture disease phenotype more completely than any individual measurement [1,10].

The clinical validation of multimodal AI models in MPUS faces several interconnected challenges. Training datasets drawn from single institutions may not generalize to different patient demographics, ultrasound system vendors, or operator skill levels, a limitation that has undermined the translational trajectory of numerous promising models [84]. Prospective multicenter validation studies with pre-specified analytical protocols and independent test cohorts represent the minimum evidentiary standard for clinical implementation, yet remain rare in the ultrasound radiomics literature [51,84]. Class imbalance, reflecting the relative rarity of malignant or advanced-stage cases in real-world clinical populations, introduces systematic bias toward the majority class unless explicitly addressed through oversampling, synthetic data generation, or cost-sensitive learning strategies [44,84]. Explainability, the capacity to render AI model outputs interpretable to clinicians, is a prerequisite for clinical adoption, as black-box predictions without mechanistic transparency are unlikely to be accepted in diagnostic workflows that carry significant patient management consequences [17,84].

Taken together, these constraints mean that the great majority of AI- and radiomics-based MPUS models published to date remain at the proof-of-concept stage rather than constituting clinically deployable tools. Three limitations are particularly consequential and warrant explicit acknowledgment. First, robust external validation on independent cohorts and on data acquired with different ultrasound systems is still the exception rather than the rule, so reported performance metrics frequently reflect optimistic, internally validated estimates. Second, radiomic features are highly sensitive to acquisition settings and to vendor-specific signal processing, and inconsistent adherence to standardization frameworks such as IBSI limits reproducibility and cross-study comparability (discussed further in Section 5.2). Third, inter-system and inter-operator variability in image acquisition introduces a domain shift that can substantially degrade model performance when an algorithm trained on one platform is applied to another. Until these issues of external validation, standardization, and cross-system generalizability are systematically addressed, the clinical role of AI-enhanced MPUS should be regarded as adjunctive and investigational rather than autonomous.

The AI-assisted multiparametric paradigm also extends beyond imaging to the broader ecosystem of clinical decision support, where the integration of quantitative biomarkers with AI-driven analysis of non-imaging clinical data has shown promising predictive performance across several medical domains, including arrhythmia risk stratification [93], precision assessment in sports medicine and youth athlete monitoring [94], and non-invasive electrocardiographic screening for early cardiovascular risk [95]. Although methodologically distinct from ultrasound-based biomarkers, these applications share the same principle of multiparametric quantitative data integration through AI, supporting its extension to MPUS-centered predictive frameworks.

## 5. Limitations, Standardization, and Future Perspectives

### 5.1. Technical and Operator-Dependent Limitations

Despite its considerable diagnostic potential, multiparametric abdominal ultrasound is subject to technical and operator-dependent constraints that must be acknowledged to contextualise its clinical applicability and guide future development. These limitations operate at multiple levels, from acoustic physics to examination workflow, and interact with patient-specific factors to determine the reliability and reproducibility of MPUS-derived biomarkers [8,22].

Acoustic access remains the fundamental prerequisite for any ultrasound-based measurement. Obesity, prominent bowel gas, post-surgical scarring, and narrow intercostal windows restrict the acoustic beam path to the target organ, leading to measurement failure or unreliable results in a non-trivial proportion of patients. Transient elastography (FibroScan) has a documented failure rate of approximately 5% and an unreliable result rate of up to 20% in patients with high body mass index or narrow intercostal spaces, with obesity representing the single most impactful patient-level confounder [8,29]. While M+ and XL probes have partially mitigated obesity-related failure, the problem persists in patients with extreme adiposity [5,28]. Shear-wave elastography techniques are less prone to outright failure but remain susceptible to measurement inflation caused by post-prandial hepatic congestion, cardiac pulsation artefacts transmitted to the liver parenchyma, acute hepatic inflammation with oedema, and operator-dependent beam angulation relative to fibre orientation in anisotropic tissues [22,27,28].

Quantitative CEUS parameters are sensitive to injection technique, cardiac output, respiratory motion during acquisition, and the timing of region-of-interest placement relative to the injection bolus, variables that introduce intra- and inter-examination variability even under controlled conditions [35,37]. Elastography measurements in the pancreas and small intestine are particularly challenging due to the depth, position, and compressibility of these organs, and the superimposition of respiratory and peristaltic motion on shear-wave propagation [71,79]. Quantitative ultrasound parameters, attenuation coefficient, sound speed, and viscosity, are subject to vendor-specific implementation differences in signal processing algorithms, transducer characteristics, and measurement software, meaning that absolute values obtained on different ultrasound platforms are not directly interchangeable without system-specific calibration [22,24,41].

Operator expertise represents a persistent source of variability in MPUS execution and interpretation. The acquisition of reliable elastographic measurements requires training in correct probe positioning, breath-hold coordination, and recognition of acquisition artefacts; CEUS interpretation demands familiarity with enhancement dynamics across multiple organ systems and lesion types; and the integration of multiparametric findings into a clinically coherent assessment requires systematic training that is not universally available [8,22,37]. These operator-dependency challenges are being progressively addressed through AI-assisted quality scoring, automated measurement guidance, and structured training programmes endorsed by EFSUMB and WFUMB, but standardized competency frameworks for MPUS remain to be universally implemented [28,35].

### 5.2. Standardization of Protocols and Biomarker Validation

The clinical translation of MPUS-derived imaging biomarkers from research tools to routine practice is contingent on achieving a level of standardization, reproducibility, and external validation commensurate with the requirements of regulated clinical decision-making. This remains one of the most critical unresolved challenges in the field [1,22,96].

Cross-vendor variability in absolute quantitative values, particularly for shear-wave velocity, attenuation coefficient, and viscosity, has historically impeded the establishment of universal diagnostic thresholds applicable across ultrasound platforms. A “rule of four” for ARFI-based p-SWE and a “rule of five” for VCTE-based LSM have been proposed as vendor-neutral interpretation frameworks for hepatic fibrosis, representing meaningful progress toward interoperability, but their extension to non-hepatic organs and to multi-parameter MPUS protocols requires further validation [28,32]. The Quantitative Imaging Biomarkers Alliance (QIBA), operating under RSNA, has provided a systematic framework for the development, performance characterization, and regulatory qualification of quantitative imaging biomarkers, including multiparametric QIB combinations, establishing statistical methodologies for the estimation of repeatability, reproducibility, and fitness for purpose that are directly applicable to MPUS parameter validation [96]. The adoption of QIBA-aligned validation standards in MPUS research would substantially accelerate the evidentiary pathway toward clinical regulatory approval.

Radiomic biomarkers derived from MPUS data face additional standardization challenges related to the high dimensionality of extracted feature sets, the risk of overfitting in small training cohorts, the sensitivity of radiomic features to image acquisition parameters (MI, frequency, depth setting, harmonic mode), and the lack of universal preprocessing pipelines. The Image Biomarker Standardization Initiative (IBSI) has established harmonized feature definitions for radiomic computation, but compliance remains inconsistent across published studies, limiting cross-study comparability and meta-analytic synthesis [9,51]. Prospective multicenter validation cohorts with pre-registered analytical protocols, external test datasets drawn from independent institutions and ultrasound systems, and transparent reporting aligned with TRIPOD-AI guidelines are prerequisites for the credible clinical translation of AI-based MPUS models [84,96].

At the level of clinical guidelines, ongoing updates from EFSUMB, WFUMB, and national ultrasound societies progressively incorporate emerging evidence for quantitative MPUS parameters into evidence-graded recommendations, a process that is inherently iterative and requires continuous alignment between research output and guideline development cycles [15,28,35]. The establishment of disease-specific MPUS protocols, analogous to the structured multiparametric MRI protocols that have achieved regulatory acceptance for prostate and liver cancer imaging, would provide the procedural standardization necessary for reproducible, widely adoptable clinical implementation [97].

### 5.3. Integration with Clinical, Biological, and Molecular Data

The greatest potential of multiparametric ultrasound imaging biomarkers in predictive and personalized medicine lies not in their isolated diagnostic performance but in their integration within comprehensive, multi-layered patient data frameworks that combine imaging phenotype with clinical context, biochemical markers, and molecular profiling [1,10]. This integration paradigm mirrors the trajectory of multiparametric MRI, where the combination of morphological, diffusion, and perfusion sequences with clinical and histopathological data has transformed disease characterization in oncology and beyond [97].

In hepatology, composite scores incorporating MPUS-derived parameters alongside serum biomarkers have already demonstrated superior diagnostic performance compared to either data source alone: the VAS-MASH-US score combining Vi.PLUS viscosity, serum AST, and sound speed achieved an AUROC of 0.75 for MASH diagnosis with 79% sensitivity [11]; sequential algorithms combining serum FIB-4 with elastographic LSM reduced liver biopsy necessity by 40–60% while maintaining high sensitivity for advanced fibrosis [56,57]. These multi-source models represent a template for the broader integration of MPUS biomarkers with clinical and biochemical data in disease-specific prediction algorithms across all organ systems amenable to multiparametric sonographic assessment.

The integration of imaging biomarkers with molecular data, genomics, transcriptomics, proteomics, and metabolomics constitutes the frontier of radiogenomics, a field that seeks to identify imaging correlates of molecular disease subtypes and to use imaging features as surrogates for molecular measurements that are difficult to obtain non-invasively at scale [2,17,18]. In HCC, MPUS-derived radiomic signatures have shown the capacity to predict TP53 mutation status and immunotherapy response, molecular endpoints with direct therapeutic implications, positioning MPUS as a potential tool for non-invasive molecular subtype assignment in clinical practice [64,65]. The prospective validation of such radiogenomic models in multi-institutional cohorts, with parallel molecular profiling and imaging biomarker extraction under standardized conditions, represents a high-priority research direction.

Personalized medicine encompasses not only biological and imaging biomarkers but also patient-reported outcomes, functional status, and psychosocial dimensions that collectively determine therapeutic tolerance, adherence, and quality-adjusted treatment benefit [98,99]. The recognition that quality of life, across domains including physical function, emotional wellbeing, and social participation, constitutes an outcome measure of equal clinical validity to survival or imaging response has reshaped oncological and chronic disease management paradigms in recent years [98,99]. Non-invasive risk assessment tools that capture biological disease phenotype through MPUS biomarkers must therefore be integrated within holistic clinical frameworks that account for patient-specific context, comorbidity burden, functional reserve, and treatment preferences [99,100]. The same quantitative principles extend beyond the abdominal organs: shear-wave assessment of diaphragmatic thickness and stiffness has been applied as an early imaging biomarker of respiratory sarcopenia in dialysis patients and in malnourished children, and core-muscle (rectus abdominis and diaphragmatic) elastography has revealed measurable alterations in paediatric functional disorders such as nocturnal enuresis. Together with musculoskeletal evaluation and growth monitoring in paediatric and adolescent populations, these applications illustrate the transversal utility of quantitative ultrasound and elastography across age groups and clinical domains, reinforcing the broader applicability of the biomarker-guided, personalized assessment approach [100,101,102,103].

### 5.4. Cost-Effectiveness, Accessibility, and Clinical Implementation

A frequently underemphasized dimension of the clinical translation of MPUS concerns its economic value, accessibility, and the practical requirements for routine implementation. The intrinsic advantages of ultrasound, namely low unit cost, absence of ionizing radiation and nephrotoxic contrast, portability, and repeatability, provide a favourable baseline for cost-effective deployment, and sequential non-invasive strategies that reduce reliance on liver biopsy, such as the combination of serum FIB-4 with elastographic LSM, have already demonstrated meaningful reductions in invasive testing and associated costs in chronic liver disease [56,57]. However, formal health-economic analyses evaluating complete multiparametric protocols (incorporating elastography, CEUS, quantitative ultrasound, and AI-assisted analysis) remain scarce, and the cost-effectiveness of MPUS relative to established cross-sectional imaging pathways has not yet been rigorously established across most non-hepatic indications.

Accessibility represents a further constraint. Several MPUS components, including 2D-SWE, viscosity imaging, quantitative attenuation tools, and CEUS with dedicated contrast-specific software, are available predominantly on mid- to high-end ultrasound systems and require ultrasound contrast agents and trained personnel, conditions that are unevenly met across primary care, community hospitals, and resource-limited settings. This heterogeneity in equipment and contrast availability risks creating disparities in access to MPUS-derived biomarkers. The acquisition of reliable multiparametric measurements also entails a substantial learning curve and demands structured operator training, yet standardized competency and certification frameworks for MPUS are not yet universally implemented [28,35]. Integration into routine workflows is additionally affected by examination time, the absence of unified reporting standards, and variable reimbursement policies, all of which influence the feasibility of widespread clinical adoption.

Finally, the current evidence base is dominated by studies of diagnostic accuracy, whereas data linking MPUS-guided clinical decisions to long-term patient outcomes, such as the durability of biopsy avoidance, prevention of disease-related complications, and effects on survival or quality-adjusted outcomes, remain limited. Prospective cohort studies with extended follow-up and clinically meaningful endpoints are required to confirm that the diagnostic gains of MPUS translate into improved long-term clinical and economic benefit.

### 5.5. Future Directions in Predictive and Personalized Medicine

The convergence of advancing ultrasound hardware, AI-powered image analysis, and precision medicine frameworks delineates a trajectory for MPUS that extends substantially beyond its current clinical role. Several directions merit particular attention as the field evolves toward a more central position in personalized abdominal medicine.

Three-dimensional and volumetric MPUS, currently available on high-end platforms for select applications, will progressively enable the acquisition of complete organ stiffness maps, volumetric fat quantification, and three-dimensional perfusion modelling, overcoming the sampling limitations of single-plane two-dimensional assessment and enabling more representative organ-level biomarker extraction [21,78]. The integration of MPUS with real-time AI analysis, where quantitative biomarkers are computed and displayed during the examination itself, rather than through post-hoc offline processing, represents the next generation of decision support tools, enabling immediate, examination-time clinical guidance without requiring specialist radiomics infrastructure [34,43,84].

Wearable and portable ultrasound devices, with AI-assisted image acquisition guidance, will extend the reach of quantitative ultrasound assessment beyond radiology departments to point-of-care settings, primary care clinics, and remote or resource-limited environments, democratizing access to MPUS-derived biomarkers for populations currently underserved by specialist imaging [84,95]. Cloud-based AI platforms enabling the centralized analysis of MPUS data acquired across distributed clinical sites will address the data aggregation challenge that currently limits the training of generalizable multiparametric models [84].

The development of longitudinal MPUS monitoring protocols, analogous to the serial elastography follow-up frameworks established for MASLD and portal hypertension, for pancreatic, renal, vascular, and intestinal disease will require prospective cohort studies with standardized acquisition timelines, clinically relevant endpoint definitions, and sufficient follow-up duration to capture disease trajectory and treatment response [5,46,61]. Regulatory pathways for AI-based medical devices, including the FDA’s Software as a Medical Device (SaMD) framework and the EU’s Medical Device Regulation (MDR), provide the evidentiary and governance structures within which validated MPUS AI tools must be positioned to achieve clinical deployment [84].

**Figure 5:**
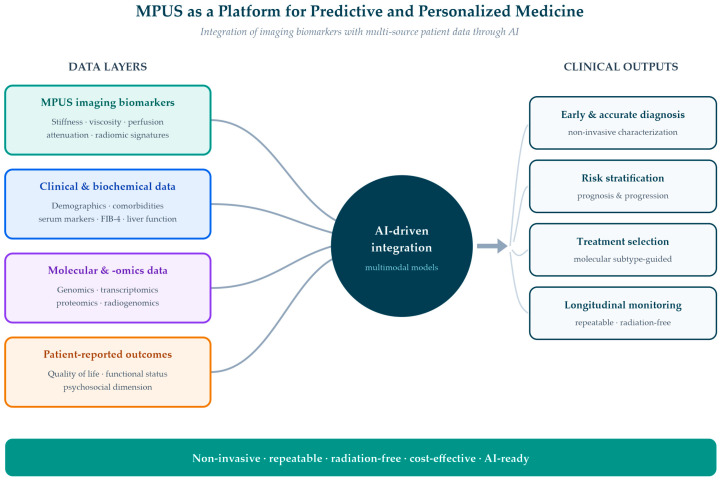
MPUS as a Platform for Predictive and Personalized Medicine.

The conceptual framework integrating MPUS imaging biomarkers within a predictive and personalized medicine paradigm is summarised in Figure 5.

Ultimately, the realization of multiparametric abdominal ultrasound as a platform for predictive and personalized medicine will depend on the sustained alignment of technological innovation, rigorous clinical validation, methodological standardization, and integration within patient-centred care pathways, a multidisciplinary endeavour requiring coordinated effort from radiologists, hepatologists, oncologists, engineers, data scientists, and regulatory bodies [1,10,17,96].

## 6. Conclusions

Multiparametric abdominal ultrasound has evolved from a conceptual framework into a clinically actionable imaging platform, capable of generating a panel of quantitative, reproducible, and biologically meaningful biomarkers within a single, non-invasive examination session. By integrating B-mode structural assessment, Doppler hemodynamic indices, elastographic stiffness and viscosity measurements, contrast-enhanced perfusion analysis, and quantitative acoustic parameters, MPUS addresses the fundamental limitation of conventional ultrasound, its predominantly morphological and qualitative character, and repositions it as a functional, data-rich instrument aligned with the requirements of predictive and personalized medicine [1,4,5].

The evidence base for hepatic MPUS applications is the most mature and clinically consequential, with validated protocols for non-invasive MASLD staging, portal hypertension assessment, focal lesion characterization within standardized reporting systems, and post-treatment response evaluation that collectively have transformed the clinical management of chronic liver disease and reduced dependence on invasive tissue sampling [5,11,12]. The expanding body of evidence for pancreatic, renal, vascular, intestinal, and soft tissue applications demonstrates that the multiparametric paradigm is transferable across abdominal organ systems, generating diagnostically relevant biomarkers wherever tissue mechanical properties, perfusion dynamics, and acoustic signatures carry pathological information [15,79].

The integration of radiomics and artificial intelligence into MPUS workflows has substantially amplified the biomarker yield of multiparametric data, enabling the extraction of high-dimensional imaging signatures that encode molecular subtype, treatment sensitivity, and long-term prognosis at a level of granularity inaccessible to human visual interpretation [2,3,17,18]. Machine learning and deep learning models trained on multiparametric ultrasound data have demonstrated diagnostic performance approaching or exceeding expert-level assessment in specific, well-defined tasks, and their continued refinement, through larger multicenter training datasets, robust external validation, and explainability engineering, will progressively bring AI-assisted MPUS into routine clinical practice [84].

The principal barriers to full clinical translation remain the absence of universal acquisition protocols, cross-vendor variability in absolute quantitative thresholds, insufficient multicenter validation of both conventional and AI-based MPUS models, and the need for systematic integration of imaging biomarkers with clinical, biochemical, and molecular data within holistic predictive frameworks [1,10,96]. Addressing these challenges requires coordinated efforts from technology developers, clinical investigators, guideline-producing societies, and regulatory agencies, converging around the shared objective of establishing MPUS as a validated, interoperable, and widely accessible platform for precision abdominal imaging.

In conclusion, multiparametric abdominal ultrasound holds genuine promise as a central imaging platform in the emerging ecosystem of predictive and personalized medicine: non-invasive, repeatable, radiation-free, potentially cost-effective, and amenable to AI-powered analysis. Its future realization in clinical practice is contingent not merely on technological advancement but on the rigorous scientific and methodological work of standardization, validation, and integration that transforms promising research findings into evidence-based clinical tools. The trajectory of current evidence supports cautious optimism that this promise will be fulfilled.

## Figures and Tables

**Table 1:** Elastography techniques.

Technique	Physical Principal	Output Biomarker	Liver Fibrosis Thresholds	Key Advantages	Main Limitations	Ref.
Transient Elastography (VCTE, FibroScan)	External 50 Hz mechanical vibration; shear wave tracked by 1D pulse-echo	Liver stiffness (kPa); CAP for steatosis (dB/m)	F ≥ 2: ~7.9; F ≥ 3: ~10.3; F4: 11.9–13.0	Extensively validated; rapid; simultaneous CAP fat quantification; standardized cut-offs	No real-time image guidance; ~5% failure, up to 20% unreliable in obesity/ascites	[8,29,30]
Point SWE (p-SWE/ARFI)	Focused acoustic radiation force push pulse at user-defined ROI	Shear wave velocity (m/s) or stiffness (kPa)	Vendor-dependent; “rule of four” framework	Integrated with B-mode; operator-guided ROI; effective in obesity/ascites	Small sampling volume; single-plane measurement; vendor variability	[27,28,31]
2D-SWE	Ultrafast plane-wave compounding tracking multiple shear fronts	Color-coded stiffness map (kPa), real-time	Superior accuracy for F ≥ 2 vs. ARFI/TE	Spatial stiffness mapping; captures heterogeneity; highest accuracy for significant fibrosis	High equipment cost; requires operator training; motion-sensitive	[26,31,32]
Strain Elastography	Relative tissue deformation under compression	Qualitative color map; strain ratio (semi-quantitative)	Not applicable (no absolute values)	No dedicated hardware; useful for superficial organs, bowel wall, lymph nodes	No absolute stiffness; compression-dependent; operator variability	[6,16]

**Table 2:** Imaging Biomarkers Derived from Multiparametric Ultrasound: Biophysical Basis, Quantification, and Clinical Application.

MPUS Component	Imaging Biomarker	Biophysical/ Biological Substrate	Quantitative Unit	Principal Abdominal Application(s)	Evidence Maturity	Ref.
B-mode	Hepatorenal index/parenchymal echogenicity	Hepatic fat infiltration	Ratio (a.u.)	Steatosis screening	Moderate	[5,11,24]
Doppler	Resistive index (RI)	Downstream vascular resistance	Ratio (0–1)	CKD progression, hepatic/splenic resistance	Established	[15,25]
Doppler	Portal vein flow velocity	Portal hemodynamics	cm/s	Portal hypertension	Established	[25]
Doppler	Limberg vascularity score	Mural hyperemia/active inflammation	Grade 0–3	IBD activity	Established	[16]
Elastography	Liver stiffness (LSM)	Fibrosis, collagen deposition	kPa/m/s	Fibrosis staging, cACLD	Established (high)	[8,27,28,29]
Elastography	Spleen stiffness (SSM)	Portal congestion, splenic remodeling	kPa	High-risk varices prediction	Growing	[45,46]
Elastography	Shear-wave viscosity/dispersion	Inflammation, edema, hepatocyte ballooning	(m/s)/kHz·Pa·s	MASH detection, inflammation grading	Emerging	[11,41,42]
CEUS	Peak enhancement (PE)	Microvascular density/blood volume	a.u.	Focal lesion characterization	Established	[2,47]
CEUS	Wash-in/wash-out rate	Microvascular perfusion kinetics	a.u./s	HCC vs. ICC, post-ablation response	Established	[23,47,48]
CEUS	Time to peak (TTP)	Vascular transit time	s	Quantitative perfusion analysis	Growing	[37]
CEUS	Adventitial microbubble uptake	Vasa vasorum neovascularization/inflammation	Qual./quant.	AAA wall biology, plaque vulnerability	Emerging	[49,50]
QUS	Attenuation coefficient (ATI/CAP)	Acoustic absorption by fat	dB/cm/MHz (dB/m)	Steatosis grading	Established	[24,30,33]
QUS	Backscatter coefficient	Tissue microstructure/scatterer density	dB	Steatosis, NASH characterization	Emerging	[39,40]
QUS	Sound speed	Tissue density and fat composition	m/s	Steatosis quantification	Emerging	[39,41]
Radiomics/AI	Texture & GLCM/GLRLM features	Sub-visual tissue heterogeneity	Dimensionless	Molecular subtyping, prognosis	Emerging	[3,9,51]
Radiomics/AI	Deep learning signatures	Composite multiparametric phenotype	Model probability	Fibrosis, malignancy, treatment response	Emerging	[19,21,52]

**Table 3:** Key Clinical Studies on MPUS in Liver Disease.

Study (Ref.)	Design/Population	MPUS Techniques	Target Condition	Key Diagnostic Performance
Cassinotto 2016 [58]	Prospective, *n* = 291, NAFLD, biopsy-controlled	2D-SWE (SSI), TE, ARFI	Fibrosis staging	AUROC F ≥ 2: SSI 0.86 > TE 0.82 > ARFI 0.77
Mózes 2022 [57]	IPD meta-analysis, *n* = 5735 (LITMUS)	TE (LSM), serum FIB-4	Advanced fibrosis (NAFLD)	Sequential FIB-4 + LSM ↓ biopsy need to ~33%; high sensitivity F ≥ 3
Pennisi 2023 [56]	Multicenter, NAFLD + T2D	TE (LSM), CAP, FIB-4	Disease severity	LSM AUROC 0.85–0.88 advanced fibrosis
Liguori 2025 [11]	Prospective, *n* = 120, biopsy-confirmed MASLD	2D-SWE, ATI, sound speed, Vi.PLUS	MASH/ballooning	VAS-MASH-US score AUROC 0.75 (79% sensitivity); Vi.PLUS AUROC 0.72 ballooning
Ainora 2023 [47]	Prospective, *n* = 82, histology-confirmed	D-CEUS (PE, WiR), 2D-SWE	HCC vs. ICC	MP-US score AUROC 0.836
Karagiannakis 2024 [45]	Systematic review + meta-analysis	2D-SWE spleen stiffness	High-risk varices	AUROC 0.87–0.91; cut-off 53.25 kPa (100 Hz)
Hirooka 2023 [46]	Prospective, CLD cohort	2D-SWE (liver + spleen), splenic volume	Portal hypertension	Integrated MPUS model > single parameters for decompensation prediction
Wang 2019 [19]	Prospective multicenter, *n* > 300, CHB	Deep learning radiomics + 2D-SWE	Fibrosis staging	AUROC 0.93–0.97 for F ≥ 2/cirrhosis
Bu 2025 [64]	Retrospective, *n* = 154, HCC	B-mode radiomics + clinical (XGBoost)	TP53 mutation status	AUROC 0.846 (>clinical-only, radiomics-only)
Lu 2025 [66]	Retrospective, external validation	B-mode + CEUS radiomics (deep learning)	Macrotrabecular-massive HCC	AUROC 0.812 (external cohort)
Zou 2025 [48]	Meta-analysis	CEUS + serum biomarkers	Post-ablation HCC response	Combined model > either alone for local progression

Note: Several listed AUROC values and cut-offs derive from single-center or small-sample studies (e.g., refs. [11,45,47]) and should be regarded as preliminary, requiring external multicenter validation before clinical adoption.

**Table 4:** MPUS Applications in Pancreatic, Renal, Vascular, and Digestive Pathology.

Organ/System	Clinical Application	MPUS Techniques	Imaging Biomarker/ Key Finding	Ref.
Pancreas	Acute pancreatitis severity	CEUS, B-mode, Doppler	Necrosis detection: sensitivity 91%, specificity 100%; CEUS vs. CT severity index r = 0.926	[68,69]
Pancreas	Solid mass (PDAC vs. non-ductal)	CEUS, elastography	PDAC hypoenhancement: sens. 90%, spec. 100%; non-ductal iso/hyperenhancement: sens. 100%, spec. 90%	[70,71]
Kidney	Renal mass (RCC vs. AML)	CEUS, Doppler, SWE	Clear-cell RCC: arterial hyperenhancement + early wash-out; papillary RCC: hypoenhancement	[72,73,74]
Kidney	CKD/renal fibrosis	2D-SWE, Doppler RI	Cortical SWE vs. fibrosis grade r = 0.61; combined SWE + eGFR ↓ biopsy; RI > 0.70 = ↓ GFR	[75,76]
Carotid	Plaque vulnerability	CEUS, 2D-SWE, color Doppler	CEUS neovascularization: sens. 87.1%, spec. 58.3%; SWE softness = instability	[49]
Abdominal Aorta	AAA wall biology/EVAR endoleak	CEUS, Doppler	Adventitial microbubble uptake in 61% AAA ≥ 4 cm (↑ inflammation); CEUS ≈ CTA for endoleak	[50,78]
Bowel (IBD)	Crohn’s activity/stricture	B-mode, Doppler, CEUS, SWE	Fibrotic vs. inflammatory stricture AUROC 0.92–0.95; CEUS normalization precedes wall thinning	[16,79]
Small Intestine	Neoplasm characterization	B-mode, CEUS, Doppler	Lesion-level risk stratification (GIST, carcinoid, lymphoma)	[81]
Lymph Nodes/Soft Tissue	Reactive vs. malignant	CEUS, Doppler, SWE	Enhancement pattern + perfusion index + vascular architecture	[82]

Note: Performance metrics for several non-hepatic applications originate from single-center or small cohorts and are presented as indicative rather than validated diagnostic thresholds.

## Data Availability

No new data were created or analyzed in this study.

## Abbreviations

AAA	Abdominal aortic aneurysm
ACR	American College of Radiology
AI	Artificial intelligence
AML	Angiomyolipoma
AP	Acute pancreatitis
ARFI	Acoustic radiation force impulse
AST	Aspartate aminotransferase
ATI	Attenuation imaging (attenuation imaging coefficient)
AUC	Area under the curve
AUROC	Area under the receiver operating characteristic curve
cACLD	Compensated advanced chronic liver disease
CAP	Controlled attenuation parameter
CE-EUS	Contrast-enhanced endoscopic ultrasound
CEUS	Contrast-enhanced ultrasound
CHB	Chronic hepatitis B
CKD	Chronic kidney disease
CNN	Convolutional neural network
CSPH	Clinically significant portal hypertension
CT	Computed tomography
CTA	Computed tomography angiography
D-CEUS	Dynamic contrast-enhanced ultrasound
eGFR	Estimated glomerular filtration rate
EFSUMB	European Federation of Societies for Ultrasound in Medicine and Biology
EVAR	Endovascular aneurysm repair
EUS	Endoscopic ultrasound
FDA	Food and Drug Administration
FIB-4	Fibrosis-4 index
FLL	Focal liver lesion
GFR	Glomerular filtration rate
GIST	Gastrointestinal stromal tumor
GLCM	Gray-level co-occurrence matrix
GLRLM	Gray-level run-length matrix
GLSZM	Gray-level size-zone matrix
HCC	Hepatocellular carcinoma
HRI	Hepatorenal index
HVPG	Hepatic venous pressure gradient
IBD	Inflammatory bowel disease
IBSI	Image Biomarker Standardization Initiative
ICC	Intrahepatic cholangiocarcinoma
IPMN	Intraductal papillary mucinous neoplasm
IPD	Individual patient data
IUS	Intestinal ultrasound
LI-RADS	Liver Imaging Reporting and Data System
LSM	Liver stiffness measurement
MASH	Metabolic dysfunction-associated steatohepatitis
MASLD	Metabolic dysfunction-associated steatotic liver disease
MDR	Medical Device Regulation
MI	Mechanical index
ML	Machine learning
MPIUS	Multiparametric intestinal ultrasound
MPUS	Multiparametric ultrasound
MRI	Magnetic resonance imaging
MRI-PDFF	Magnetic resonance imaging-derived proton density fat fraction
MTM	Macrotrabecular-massive (hepatocellular carcinoma subtype)
MTT	Mean transit time
NAFLD	Non-alcoholic fatty liver disease
NASH	Non-alcoholic steatohepatitis
PanNEN	Pancreatic neuroendocrine neoplasm
PCL	Pancreatic cystic lesion
PDAC	Pancreatic ductal adenocarcinoma
PD-1	Programmed cell death protein 1
PE	Peak enhancement
PHQ-9	Patient Health Questionnaire-9
PI	Pulsatility index
p-SWE	Point shear-wave elastography
QIB	Quantitative imaging biomarker
QIBA	Quantitative Imaging Biomarkers Alliance
QUS	Quantitative ultrasound
RCC	Renal cell carcinoma
RF	Radiofrequency
RI	Resistive index
ROI	Region of interest
RSNA	Radiological Society of North America
SaMD	Software as a Medical Device
SF-36	36-Item Short Form Health Survey
SPL	Solid pancreatic lesion
SSI	Supersonic shear imaging
SSM	Spleen stiffness measurement
SVM	Support vector machine
SWE	Shear-wave elastography
T2D	Type 2 diabetes
TACE	Transarterial chemoembolization
TE	Transient elastography
TIC	Time–intensity curve
TIRADS	Thyroid Imaging Reporting and Data System
TP53	Tumor protein 53 (gene)
TRA	Treatment Response Assessment
TRIPOD-AI	Transparent Reporting of a multivariable prediction model for Individual Prognosis Or Diagnosis–Artificial Intelligence
TTP	Time to peak
UCA	Ultrasound contrast agent
US	Ultrasound
VAS-MASH-US	Viscosity-AST-Sound speed MASH ultrasound (composite score)
VCTE	Vibration-controlled transient elastography
VEGF	Vascular endothelial growth factor
WFUMB	World Federation for Ultrasound in Medicine and Biology
WiAUC	Wash-in area under the curve
WiR	Wash-in rate
2D-SWE	Two-dimensional shear-wave elastography
3D	Three-dimensional
